# Value-added-tax rate increases: A comparative study using difference-in-difference with an ARIMA modeling approach

**DOI:** 10.1057/s41599-023-01608-y

**Published:** 2023-03-21

**Authors:** Mahfoudh Hussein Mgammal, Ebrahim Mohammed Al-Matari, Talal Fawzi Alruwaili

**Affiliations:** 1 Accounting Department-College of Business, Jouf University, Sakaka, Saudi Arabia; 2 Amran University, Amran, Yemen

**Keywords:** Business and management, Economics, Finance

## Abstract

Collecting and managing taxes is a critical keystone to protecting a country’s financial intensity and developing a country’s tax system, where value-added tax (VAT) has proven to be a nurturing and steady foundation of income for governments. VAT is a primary source of financial gain in developing nations, which differs from economic income in developed nations, where economic income is primarily derived from tax income. This study examines the influence of the new VAT on non-financial Saudi-listed companies. We compared and examined the impact of the newly introduced 15% VAT in various non-financial sectors. Using charts, tables, appropriate variance analysis, and ARIMA model in the Difference in Difference approach. Regarding data, we targeted 2019 before the new VAT, the discovery of COVID-19, and 2020 after the new VAT and during the COVID-19 pandemic. Results show that a sharp increase in VAT (with other unmeasured variables in this study) has a significant positive/negative impact on inter-industry volatility in corporate financial reporting metrics and was even more dramatic with the COVID-19 disaster. The proposed 10% VAT increase has caused significant fluctuations in companies’ metrics, for example, a decrease in average profits—2.16%, an escalation in more inactive companies, and a higher probability of bankruptcy among companies. Such an outcome would undoubtedly affect unemployment and possibly lead to a reduction in tax revenues in the long run. The consequences of an unexpected VAT increase raise the question of whether they accurately reflect the natural tax system in Saudi Arabia. Therefore, the Saudi government may consider the practical implications of outcomes before taking the next step in interventions in non-financial sectors.

## Introduction

VAT, a broad-based expenditure tax, has been relatively new among the government’s most essential revenues (Adhikari, 2020). Since the introduction of VAT in over 170 nations, it typically increases by 20% or more of all tax revenues (Mgammal, 2021). Sixty years ago, VAT was rarely taught in certain professional texts from outside “France”. Other tax frameworks have many problems. Some nations have similarly employed a VAT introduction to diminish other ineffective implicit taxes, for example, stamp duties, excise taxes, and tariffs, or to somewhat decrease direct taxes, e.g., the company income tax rate and personal income tax rate. Nevertheless, the developments were contrasted slightly with VAT’s substitution of sales taxes (Adhikari, 2020).

With its 2030 vision, the Kingdom of Saudi Arabia (KSA) allows the government to be resilient and not rely solely on oil revenues. In contrast, it will become a burden to a lesser extent for the end customer as it is the first type of tax bracket presented in KSA. Widely implemented in sub-Saharan Africa, the Middle East, and elsewhere, it has been the cornerstone of tax improvements in numerous developing nations (Keen and Lockwood, 2010). In KSA, the General Department of Taxation and Zakat 2017 discussed that developing a tax system to support Saudi Arabia’s transformation is more in line with the new global strategy. VAT was officially introduced in early January 2018 at an average rate of 5% (General-Authority-of-Zakat-and-Tax, 2017). So far, KSA has familiarized itself with VAT by reducing its dependence on oil savings because of the COVID-19 epidemic on the planet. KSA is one of the countries making some changes to ease COVID-19 results’. In early July 2020, KSA augmented its VAT from 5% to 15% (General-Authority-of-Zakat-and-Tax, 2017). As a result, one of the purposes of this study is to quantify the effects of this unexpected increase.

Recently, Alhussain (2020) highlighted the effect of VAT enforcement on Saudi banks. Results show a slight reduction in total liabilities, assets, current accounts, and customer deposits. In addition, retained earnings and total operating costs after implementing 5% VAT also saw a significant reduction. Furthermore, there is a small growth in overall operating income and a substantial increase in net operating income after implementing 5% VAT. In addition, aside from benefits held after and before 5% VAT, there are no genuinely significant differences between the various factors being studied. Our paper looks into what happened after Saudi Arabia imposed a 15% VAT on non-financial institutions. In that way, Bogari (2020) has focused on studying the economic and social effects of implementing 5% VAT in KSA. Results display that the employment of 5% VAT escalated the monetary assets of the territory. Likewise, they perceive undesirable social effects and experience some difficulties.

As one of the GCC^1^ countries, Saudi Arabia’s tax system is regressive in taxation. Ezenagu (2021) suggests that GCC countries expand their tax rules to diversify their income and improve representation. Corporate taxes in GCC remain low as a result of foreign investors’ competition. Indirect taxes such as VAT are imposed gradually and carefully to avoid a regressive effect or discourage foreign investors from investing and living in the countries (Ezenagu, 2021). Nevertheless, VAT may be regressive rather than progressive as an indirect consumption tax. They can get more money from people with lower incomes through regressive taxation. “Progressively” means placing a tax burden on those with higher incomes (Seligman, 1893). In a recent OECD paper, Thomas (2020) noted that in the vast majority of OECD countries, VAT was either “roughly proportional” or “slightly progressive”. These are highly unpredictable, with the most significant impact on VAT’s regressive nature (Caspersen and Metcalf, 1994; Tamaoka, 1994; Carroll and Shabana, 2010).

Accordingly, it is recommended that the nature of the implementation of the VAT-exempt rule be redesigned by enhancing the competence and viability of workers in the General Office of Zakat and Taxation and streamlining tax systems for financially beneficial results and community-side returns. The other motivation for conducting this study is to explore the impression of a new 15% VAT implementation on non-financial companies listed in the Saudi stock market. This study contributed to VAT and taxation literature as one of the few to investigate VAT impact in KSA. To our knowledge, an additional incentive to complete this article is the absence of existing studies observing the effect of increasing VAT to 15% among non-financial companies listed in Saudi Arabia. This article relates to empirical studies estimating the influence of differential VAT over time. The relationship is closer to articles that explore economic efficiency from different perspectives. However, previous studies have not examined the role of VAT alone or, essentially, whether the design of how taxes are created affects growth.

Therefore, to present the issues that our study aims to point out and to mention the research gap that our study seeks to fill, our main contribution is to depend on further disaggregated data and employ a similar strategy—using a group of Saudi-listed companies as one of the G20, OECD, and GCC countries for two periods. Using charts, tables, appropriate variance analysis, and Difference in Difference, with ARIMA modeling, authenticate an underlying link between the two periods under investigation. Thus, this study considers the following question: Does the imposition of 15% VAT affect the following items? Total assets, total equity and total debt and equity, total income, total revenue, total cost and net income, and change in operations. activity, investment act change, ending cash, and endowment act change. When assessing the effects of a VAT increase on companies, the items mentioned above are included in the evaluation models to control for a potential impact of a VAT increase. Any of these items contains an area that could be affected by the VAT increase. However, a VAT increase may impact other non-determined areas.

Our results confirm that the employment of VAT in KSA has received some attention. Thus, this article clarifies a significant policy impact that may be involved for KSA and similar to that of other GCC nations considering extensive VAT rate restructuring. Overall, our findings show that an increase in the VAT rate in KSA significantly negatively impacts corporate profitability in the short term compared to other indicators and that abandoned reasons can exaggerate the magnitude of an impression. Tax increases are anticipated to boost firm profitability in the long run. Because it raises government revenues, the government increases investments which can grow businesses or social targets such as shrinking economic variations. Still, the tax itself is not anticipated to expand corporate profitability. This article offers an empirical and theoretical ground for comprehensive investigations of the consequences of VAT policy, especially the government’s refinement of tax reform strategy to encourage more investment. That would reduce unemployment and probably lead to a relief in tax revenues in long run. Therefore, the Saudi government may consider the practical implications of outcomes before taking the next step in interventions in non-financial sectors.

The lean part of the article is arranged as follows. Section “Review of related literature” is the review of associated literature, section “Methodology” is the study design, section “Results analysis and discussions” is the analysis and discussion results, and section “Conclusion and remarks” is the conclusion.

## Review of related literature

A fundamental taxation principle is a capability to pay progressive taxation, which means that high-income individuals should be taxed more heavily than low-income individuals (Smith, 1776). When VAT has a regressive influence, it undermines the goal of redistributing wealth from the wealthy to the unfortunate and worsens low-income living standards. That describes why several nations with VAT governments implement a list of exceptions on services and goods or nil-rate VAT on essential services and goods, which is mandatory for all (Cnossen, 1992). Several countries exclude the application of a VAT rate or a higher rate than the introductory rate for items that qualify as extravagance goods to ensure tax fairness, as guaranteed by the solvency principle. Hence, when GCC nations seek to implement or reform VAT systems, they must ensure that the system is not regressive and does not further impoverish low-income households (Alavuotunki et al., 2019). Dedicated to the difference in the incomes of workers who qualify as immigrants and those who qualify as expatriates, VATs can be securely applied to high-quality tourist activities, hotel accommodations, and additional services and goods (Carlson and Patrick, 1989). The VAT system is gradual in this method as it targets individuals with higher-than-average national revenues (Ezenagu, 2021).

In an inclusive, neutral, and operational way, VAT has been designed for production chain taxation and individual consumption. During the next decade of the twentieth century, VAT remained the world’s most extensive tax reform and was a key source of government revenue. VAT is extensively employed in developing and developed countries (Schenk et al., 2015). However, the spirit of VAT is that it is charged to all businesses and that its existence is attributed to tax payable on traders’ sales. It is ultimately applied if the trusted chain is stable and whether it is correctly collected from imports and sent for export only at last consumption (Keen and Lockwood, 2010).

Tax rules are expanding to some extent and are isolated from the critical reasons for practicing the synthetic control technique to measure its capability to account for unnoticed heterogeneity. They are even more acceptable than pairing techniques or old panel regressions. The framework of VAT implementation offers a unique exogenous set for assessing the impression of tax-related improvements in economic efficacy (Adhikari, 2020). VAT rule applies to most personal consumption by individuals, e.g., measured by the value paid for services and goods. The introduction of VAT helps exports since exported goods are exempt from tax. Aside from the benefit of tax exemption at the end, it is possible to recover all of the tax paid during the preceding export stages. Thus VAT contributes to the accessibility of local goods in foreign markets. Additionally, measures of VAT compliance rates are appreciated to classify VAT enforcement problems (Giesecke and Tran, 2012; Adhikari, 2020).

Prasad and Deng (2009) verify the argument of others that the United States (US) has higher progressive taxes than the *European Union (EU)*. Nevertheless, they found that the UK’s tax construction was further regressive than mainland welfare territories. Prasad and Deng (2009) show that it is wrong to presume that property taxes and taxes on income are continuously progressive. In this case, a property’s taxes are almost always regressive, and regressive taxes are considered in statistics. Payroll taxes tend to be regressive, but we see an increasing payroll tax in two cases. The only tax that keeps its status alive is the sales tax, which is regressive each year in every nation (Prasad and Deng, 2009).

Nevertheless, there are variances between Prasad and Deng’s (2009) results and those of other researchers. For example, Piketty and Saez (2007) discover superior progressivity in the US solitary in the 1970s, and Prasad observes that it remains so today. Prasad was suspicious that this inconsistency arose due to Prasad and Deng’s (2009) elimination of VAT. They are confident that the elimination of VAT and other sales taxes will misrepresent the tax’s progressivity and will be an impediment to the technique of calculating quantity progressivity using tax income.

Financial source of welfare territories in the US, which adopted tax income as its primary source of funding earlier in the First World War and repeatedly resisted VAT (Morgan and Prasad, 2009). Kato (2003) claims that nations can improve significant welfare territories only if they accept regressive taxes first. Does the regressive nature of excise taxes such as VAT matter in this context? Roughly, economists have claimed that it is not because while VAT is regressive when considered at one spot in time, it appears proportionate when viewed over a lifetime.

Caspersen and Metcalf (1994) find that VAT would be generally proportionate in the US concerning lifetime income. A similar occurrence of expenditure taxes in another country would result in a less regressive overall tax image in countries with high VAT. In contrast, Graetz (2005) presented two other disagreements in the contradiction of consuming lifetime income as a measure of VAT progressivity. Initially, young individuals’ ability to borrow in contradiction of forthcoming or lifetime income is restricted in rehearsal. This is contingent on elements, for instance, credit regulation and the risk aversion of capital markets and individuals. Subsequently, the lifetime incidence sum assumes that the VAT rate will be steady throughout the lifetime, but continuity in the rule cannot be assured.

Moreover, Caspersen and Metcalf (1994) assume that VAT alone does not modify forms of income movement. Nevertheless, a tax levied on folks when they are poor and young can have penalties that mislead income mobility throughout their lives. In the American setting, a VAT would have additional effects than in the EU setting, as it would restrict the capability of deprived households to overcome financial barriers produced by health care costs. It would harm the identical income movement that is the focus of Caspersen and Metcalf’s (1994) analysis. Because of these explanations, Prasad and Deng (2009) assume that the lifetime occurrence viewpoint could be more suitable for investigating VAT progressivity and analyzing its effects.

External forces frequently influence VAT options, seemingly due to the internal circumstances of restructuring nations. Several academics and non-academic institutions characterize the work of multi-faceted associations, particularly the “EU and International Monetary Fund (IMF)”, in affecting the increased selection of VAT. When entering the EU, states are required to implement VAT. Any country that becomes a member of the *EU* or is trying to enter the *EU* wishes to implement VAT (Keen and Lockwood, 2010; Adhikari, 2020). Likewise, *IMF* remains a staunch supporter of VAT and frequently employs stand-alone VAT practices to support and finance mortgages. Accordingly, a small nation seeking *IMF* support will likely enforce VAT (Keen and Lockwood, 2010). For example, once a country is included in *IMF’s* agenda, the probability of applying VAT rises to 25% per year. Ebrill et al. (2001) estimate that all nations familiar with VAT through the 1980s and 1990s utilized *IMF*, at least in part, based on the guidance in VAT application (Keen and Lockwood, 2010; Ufier, 2014; Čížek et al., 2017).

VAT is an indirect tax in the tourism industry, just like sales tax in the US, levied on tourism-related services and goods based on value added at various phases of production. Small restaurants, cafes, and hotels in the *EU* (HOTREC, 2020) accumulate VAT for governments (Kristjánsdóttir and Remoaldo, 2019). VAT can be seen as a macroeconomic tool for tourism management, but at the same time, it upsurges tourists’ prices. The main findings demonstrate that increased VAT does not affect traveler flows. This suggests that, as a macroeconomic instrument, governments maintain targeted VAT growth in their plans. As well as consider creating the necessary structure to accommodate the influx of tourists (Kristjánsdóttir, 2020).

The VAT construction plan in a single locality impacts the construction of the evaluation in nearby regions. If nations introduce VAT because of their neighbors’ impact, at that point, this modification tax will be progressively external to financial preconditions of implementing nation, rather than the change being convinced by their internal economic situation. The presentation of VAT undoubtedly reflects the monitored area (Case et al., 1993; Besley and Case, 1995). For example, more than 11 *EU* nations received VAT within five years of *France* choosing to implement VAT. Correspondingly, 11 additional “Latin American” countries presented VAT when Brazil opted to implement it. Mimicry is particularly common in *Eastern Europe*, where 80 nations have implemented VAT within five years of “*Hungary”* implementing VAT. A comparative example is likewise found in the generating nations of Asia and sub-Saharan Africa. Impressionist behavior in VAT appropriation is also reflected in increasingly methodical research (Keen and Lockwood, 2010; Ufier, 2014, 2017; Čížek et al., 2017).

Concerning microenterprises (new-energy industry), Sun et al. (2020) conducted a semi-general analysis to investigate the impression of new energy VAT incentives on the microenterprise level. Using the difference in difference (DiD) method to analyze data of China’s new energy-listed institutions, they found that in 2008, China’s VAT engine for new energy sectors could have been more effective in promoting return-on-equity of the company. The study recommends supporting tax incentives for using terminals to reduce overcrowding in the new energy sector. Economic dynamics must be understood to avoid the new energy sector’s changing markets and defiant struggles. Encouraging R&D incentives is a favorable condition for improving renewable energy technology. A comparison of support measures to improve the new energy sector must be made for listed Chinese companies (Sun et al., 2020).

Under the VAT approach, capital income would only be taxed once it was finally consumed (United States. Congress. Senate. Committee on, Housing, and Urban, 1992, p. 157). According to Golob (1995), VAT has an optimistic impact on stock prices. This type of tax would not take into account taxes on capital gains and dividends, but stock prices would rise for hardly fewer companies than other taxes would tax. The reason is a reduction in available income for taxation. An example of the impact of VAT is what Asogwa and Nkolika (2013) found in their research on the impact of VAT in Nigeria, which involved stocks. They discovered an optimistic effect of VAT on stocks and investment in general.

Practically all nations have introduced VAT to substitute sales taxes (i.e., manufacturing tax, wholesale tax, or sales tax). In some nations, they have similarly employed a VAT preamble to diminish more ineffective indirect taxes or, to some extent, reduce direct taxes. That also applies as a rule, but only if the nations are canvassed (Ufier, 2017). For example, in a sample of 88 nations from which the author collected information, *Panama and Japan* are the two main countries that did not have extensive sales tax before the misappropriation of VAT. *Japan* ultimately reduced the various extraction taxes, such as car sales tax, from 23% to 6%, and *Panama* diminished numerous stamp duties after they presented a value-added tax. Those VAT changes thus deliver a perfect set for assessing the influence of changing sales and sales taxes via VAT on commercial performance (Adhikari, 2020).

For example, in measuring the effects of VAT on China’s export performance, the outcomes recommend that a 1% drop in the export VAT leads to a seven-point, two-per-cent increase in eligible export values. This influence is due primarily to an alteration of quantities and several foreign markets served, whereas the median unit value of exports remains unaffected (Gourdon et al., 2022). Furthermore, evidence from listed companies in China reveals that converting business taxes to VAT has encouraged organizations to expand their innovation output and practical innovations. In addition, the level of R&D investment occupies a middle ground between the association between commercial tax changes to VAT and the overall innovation output and the association between corporate tax changes to VAT and the applicable innovation output of firms (Cao et al., 2022).

In Indonesia, Irawati et al. (2022) findings show that “e-invoice” employment and taxable personal obedience significantly positively impact VAT incomes. They recommend for Pasar-Rebo-Jakarta’s key tax office continue advancing its service in providing info and conducting campaigns related to issuing e-invoices to taxable individuals to improve compliance. Similarly, rising tax revenues can have an impact on VAT receipts. Moreover, the results of Kim et al. (2022) study in South Korea expose that trust in the government significantly strengthens consumer cooperation. They also find that stronger trust influences supportive VAT compliance when the discount amounts increase. In Asia, Permadi and Wijaya’s (2022) study results of 19 countries’ samples show that implicitــtariffs, service sector, and government efficacy significantly positively impact VAT receipts, whilst import volumes have a substantial negative impact. In contrast, standard rates, fiscal shortages, effectiveness, control of corruption, the rule of law, and the rule of democracy have no substantial impact on VAT receipts.

### Tax system in Saudi Arabia

Currently, Saudi Arabia’s tax system has been established by a series of laws and regulations, which can be classified into three main categories: tax policy (tax law), tax administration (fiscal law), and tax collection (administrative law). These policies have laid the foundation for regulating taxes in Saudi Arabia since 1962. The first law was enacted in 1962, entitled “General Principles on Taxation and Regulation of Fiscal Matters.” In 1974, another law entitled “Principles of Taxation in the Kingdom” was passed. Both laws were later replaced with two other pieces of legislation in 1984: the Law of Taxes on Income and Wealth Tax (“Law on income and wealth tax”) and the Law of Taxes on Consumption Goods (“Law on consumption goods tax”). All four of these laws are still in force today, making up the backbone of the present tax system in Saudi Arabia.

Saudi’s income tax law prohibits tax evasion in general. According to these provisions, evaluators are authorized to re-characterize transactions with no economic influence, have a legal form that does not reflect their true economic nature, or were concluded to obtain a tax advantage. There are several different taxes in place in Saudi Arabia, including VAT. With some exclusions, corporations domiciled in Saudi Arabia are subject to two distinct tax regimes based on the nationality of their owners. Income tax is levied on resident corporations held indirectly or directly by non-Saudi or GCC people. Resident firms wholly owned or controlled by Saudi or GCC citizens are only subject to Zakat. Resident corporations held jointly by Saudi Arabia or GCC and foreign residents are liable to Zakat in percentage to Saudi or GCC ownership and income tax in percentage to foreign ownership (KPMG, 2021).

If a foreign partner in an autonomous joint venture resumes activity through a permanent location in KSA, a foreign participant may be subject to KSA income tax. Profits related to a permanent establishment will be taxed in KSA. Otherwise, if the unincorporated joint venture does not result in the construction of a permanent establishment for a foreign participant, a foreign participant’s revenue from providing services to KSA clients may be subject to withholding tax (KPMG, 2021).

Saudi Arabia’s Vision-2030 contains the formation of a VAT. Hosting a VAT creates welfare that grows by up to 4.3% of present consumption (Blazquez et al., 2021). In Saudi Arabia, VAT has an important influence on the inclusive tax system. Moreover, VAT has an average effect on stock prices. As a register of financial exchanges, it measures the general action and execution of every stock in the securities market (Burton and Brown, 2014). Financial exchanges usually have a primary index that counts any tax activity that can be influenced by any external or internal component and clarifies how top organizations in the economy are acting. Also, the stock market can get extremely far from the course of the economy. Mgammal (2021) finds that organizations are, on average, less profitable after a VAT rate hike. The levied 10% VAT rate increase has produced, on average, a −2.1% reduction in profitability for Saudi’s listed organizations. Moreover, government debt had an unfavorable impact on organizations-profitability in 2020, which will threaten Saudi organizations’ long-term growth, and they have proposed several VAT incentives in the tax system.

In this context, like the Dow Jones or S&P 500, the main index of the Saudi stock exchange is the “Tadawul All Shares Index” (TASI). TASI measures the “Saudi Stock Exchange” and can track the status of each stock. Stocks typically clarify how a company behaves based on its income and sales, which guides to a respectable link between stock prices and spending levels. Meanwhile, stock prices were expected to be affected by VAT, and taxes were constantly affecting the economy. It is another impetus for this study, which aims to assess public perceptions of implementing a new 15% VAT on non-financial companies listed in Tadawul.

**Fig. 1:**
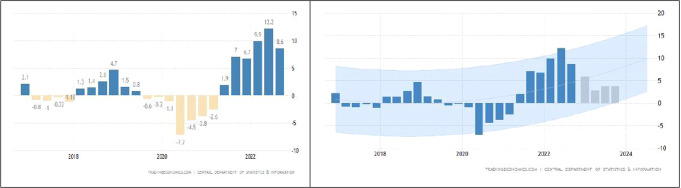
The Gross Domestic Product (GDP) in Saudi Arabia Source: (Trading Economics, 2022).

The economy is the primary driving force behind Saudi Arabia’s social and cultural advancement. Several economic sectors, including transportation, manufacturing, and services, have attracted foreign investments due to the country’s recent rapid economic expansion. Saudi Arabia’s economy ranked sixth in the world in terms of gross domestic product in 2017. Saudi Arabia’s GDP increased by 5.9% year over year in 2017. Pursuing a 12.2% rise in the previous quarter, which was strongest in the third quarter of 2011, KSA’s GDP expanded by 8.6% year on year in the third quarter of 2022. The economy grew for the sixth quarter in a row, with a 14.5% increase in oil activity and higher oil prices. Non-oil activities increased by 5.6%. The GDP of KSA improved by 2.6% on a seasonally adjusted quarterly basis, following a 2.2% increase in the previous quarter. Trading Economics global macro models and analyst forecasts state that Saudi Arabia’s annual GDP growth rate is expected to be 5.80% by the end of this quarter. Consistent with our econometric models, Saudi Arabia’s annual GDP growth rate is expected to be about 3% in 2023 (General Authority for Statistics, 2021, 2022). For more info, see Fig. 1 (Source: Trading Economics, 2022).

## Methodology

### Sampling and data sources

The comparative analysis by industry and DiD methodology are used for the study. An analysis of the time series of the data followed this. We were worried about the endogeneity of the anticipated tools for studying independent variables (IVs). So, log variation in VAT has introduced numerous suggestions for techniques to adjust the standard DiD approximation model to address this endogeneity. The average model is utilized to denote weakly stationary-stochastic time series in multinomials. This model is regularly deliberated as ARMA-(*p*,*q*)-model. We developed “ ARIMA *p*, *d*, *q* model”, demanding the variance ARIMA model consistent with AIC & BIC most minor data standards to control specific values of *p* and *q*. ARIMA is appropriate for one-dimensional models with time-dependent instabilities. The diagnostic checks conducted included the data and logarithms for the first-difference test to stabilize the change in the difference series. Augmented Dickey–Fuller (ADF) test was used to discover appropriate values of *p* in autoregression and q in moving average (MA) in the research model. ARIMA 1,1,0 model in time series investigation works well with time delay forecasting. Finally, we conducted the C–H test to distinguish autocorrelation.

In general, the experimental study of the economic impacts of VAT is scandalously irregular. It tends to be divided into diminished structure analysis and general equilibrium analysis. The primary preferred position of the general equilibrium display is that it gives distance and a clean line between the hypothetical and practical features of the fiscal policy inquiry. Though, they likewise have significant obstacles. For example, they build robust and off-the-cuff suspicions about model utility types, adaptability types, tax treatment, market structure, innovation types, etc., which are usually not maintained in reality (Adhikari, 2020).

Regarding the effect of VAT on overall economic growth, all published papers used statistics from high-income nations such as the US, Canada, Germany, and Norway (Ballard et al., 1987; Piggott and Whalley, 2001; Boeters et al., 2010; Bye et al., 2012). They found an optimistic effect of VAT on economic efficacy and other macroeconomic factors. However, when flaws arising from VAT reality are factored into the study model, the effectiveness of VAT implementation is significantly reduced. For example, Bye et al. (2012) found that including selective services in the VAT, base decreases prosperity. Similar to the non-inclusion of particular facilities in VAT rule or the inclusion of all installations in VAT source (Ballard et al., 1987).

Before applying the new 15% VAT, we used a periodic dataset from most Saudi registered companies in this article for the second and third quarters of 2019 and the third and fourth quarters of 2020. The exemplary framework was chosen when the new 15% VAT was introduced because of public access to information about VAT for non-financial companies. Tadawul requires all publicly traded companies to publish their financial statements on the Tadawul website quarterly and annually. TASI consists of 192 widely traded corporations divided into 11 main sectors. The most prominent segments are consumer discretionary, information technology, energy, consumer essentials, materials, healthcare, industries, communications services, real estate, utilities, and finance. Financial companies were excluded from the sample framework as they have particular transactions, and some previous studies have investigated the effects of VAT on KSA banks.

### Study model

The methods used in this study are clearly explained and rigorously analyzed by referring to good literature. In general, the method starts by analyzing the time series of the data to highlight how the model generates and understands the research framework. We applied comparative analysis through DiD method. Concerns about the endogeneity of the expected instrument of the variable studied, i.e., the logarithmic change in VAT, have led to various techniques being proposed to adapt standard DiD approximation models to deal with this endogeneity. Therefore, the average model is used as a polynomial as a weakly stationary random time series; this model is also known as ARIMA (*p*, *q*) model, and ARIMA is suitable for one-dimensional models with time-dependent instability.

**Fig. 2:**
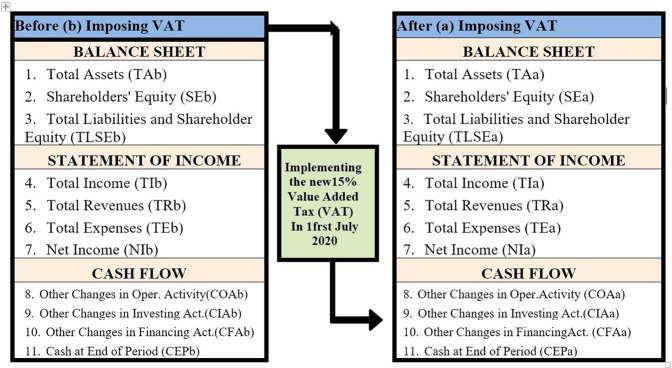
Study framework.

We use DiD approach to empirically investigate the “cost-increasing” influence of increasing VAT rates. DiD approach requires data from control and treatment groups in two different periods, precisely as in our study, one time before and one time after the introduction of “15% VAT”. Based on the practice of (Gruber and Poterba, 1994; Zou et al., 2019; Liu et al. 2022), our DiD model is reputable as follows:

$$\begin{array}{l}Y_i\left( {{\rm {treatment = arima}}\,{\rm {DID}}} \right) = \beta 0 + \beta 1TAb + \beta 2TAa\, \\ + \,\beta 3TAb\, \ast\, TAa + \beta 4SEb + \beta 5SEa + \beta 6SEb\, \ast\, SEa\, \\ + \,\beta 7TLSEb + \beta 8TLSEa + \beta 9TLSEb\, \ast\, TLSEa + \beta 10TIb\, \\ + \,\beta 11TIa + \beta 12TIb\, \ast\, TIa + \beta 13TRb + \beta 14TRa\, \\ + \,\beta 15TRb\, \ast\, TRa + \beta 16TEb + \beta 17TEa + \beta 18TEb\, \ast\, TEa\, \\ + \,\beta 19NIb + \beta 20NIa + \beta 21NIb\, \ast\, NIa + \beta 22COAb\, \\ + \,\beta 23COAa + \beta 24COAb\, \ast\, COAa + \beta 25CIAb + \beta 26CIAa\, \\ + \,\beta 27CIAb\, \ast\, CIAa + \beta 28CFAb + \beta 29CFAa + \beta 30CFAb\, \\\, \ast \,CFAa + \beta 31CEPb + \beta 32CEPa + \beta 33CEPb\, \ast\, CEPa + \varepsilon _i\end{array}$$

where *Y*_*i*_ is a linear function of the treatment, *ε*_*i*_ is the error and other variables definitions in Fig. 2 study framework.

We compared the new 15% VAT effect in different types of non-financial firms in KSA. We used the empirical DiD approach to study the influence of the new 15% VAT imposed on non-financial Saudi-listed corporations. We focused on the second and third quarters of 2019 before introducing a new 15% VAT, before the discovery of COVID-19, and on the third and fourth quarters of 2020 after introducing a new 15% VAT and during the COVID-19 epidemic. We discovered that VAT increases affect businesses both before and after the imposition of VAT but at different levels. When measuring the effect of VAT, we used an index containing 11 items: BALANCE SHEET^2^: Total assets, total equity, and liabilities-Equity. STATEMENT OF INCOME^3^: Total Income, Total Revenues, Total Expenses, and Net Income. CASH FLOW: Changes in Operation. Activity, Changes in Investing Act, Changes in Financing Act, and Cash at End of Period.

## Results analysis and discussions

The descriptive statistics for each variable before and after increasing VAT and discovering COVID-19 are shown in Table 1. From the mean perspective, the average of all financial statements’ key indicators showed an apparent decline after increasing VAT and discovering COVID-19, which aligns with our hypothesis.

**Table 1:** Variables statistics summary.

Before (b) Imposing VAT and COVID-19					After (a) Imposing VAT and COVID-19				
Variable	Mean	Std. Dev.	Min	Max	Variable	Mean	Std. Dev.	Min	Max
TAb	1.28E + 07	5.08E + 07	62,994	4.77E + 08	TAa	1.28E + 07	5.17E + 07	43,478	5.03E + 08
SEb	4,788,402	1.69E + 07	55,457	1.69E + 08	SEa	4,652,292	1.66E + 07	14,494	1.65E + 08
TLSEb	1.28E + 07	5.08E + 07	62,994	4.77E + 08	TLSEa	1.28E + 07	5.17E + 07	43,478	5.03E + 08
TIb	331,645.6	1,109,441	−3407	8,375,361	TIa	318,024.6	1,206,166	−242,725	8,970,955
TRb	347,839.8	1,140,208	−105,027	8,699,826	TRa	338,827.1	1,254,699	−178,025	9,083,262
TEb	233,783.7	980,947.3	−1,374,325	7,746,953	β17TEa	225,897.5	996,286.7	−1,199,752	7,762,214
NIb	78,985.93	356,338.2	−724,512	2,679,387	NIa	62,371.49	342,734.2	−609,803	2,765,537
COAb	55,342.46	970,096.6	−4,252,574	9,451,664	COAa	86,007.48	600,282.1	−1,329,988	6,184,696
CIAb	191,045.9	107,8954	−455,858	7,620,294	CIAa	15,994.71	268,631.9	−1,140,654	1,589,257
CFAb	−642,943	2,665,571	−2.03E + 07	517,795	CFAa	−439,313	1,907,536	−1.57E + 07	278,246
CEPb	698,759	3,357,006	−4764	3.74E + 07	CEPa	619,081.7	2,733,571	−34,220	3.02E + 07

The descriptive statistical outcomes for Financial statements’ key indicators variables before and after increasing VAT and discovering COVID-19 are shown in the table. All the numbers are in thousands of riyals (000). *Data source*: Companies’ annual reports author’s calculation.

### Comparative analysis by industry

In this context, we compared the results of financial statements’ key indicators, namely: Total Assets (TA), Shareholders’ Equity (SE), Total Liabilities and Shareholder Equity (TLSE), Total Income (TI), Total Revenues (TR), Total Expenses (TE), Net Income (NI), Other Changes in Oper. Activity (COA), Other Changes in Investing Act. (CIA), Other Changes in Financing Act. (CFA) and Cash at End of the Period (CEP). The industry classification is based on Saudi Stock Exchange Tadawul, and we excluded financial companies.

**Fig. 3:**
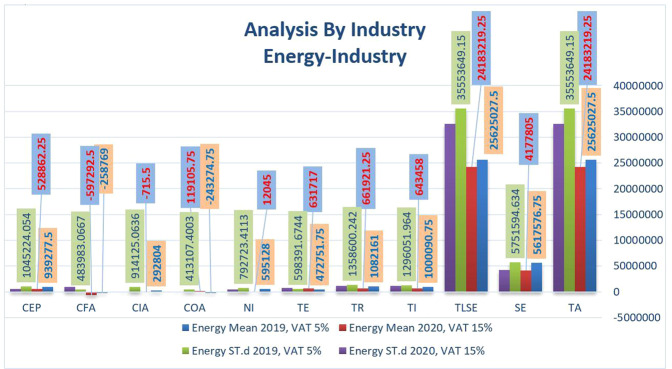
Comparison of the financial statements’ key indicators in term of the average and standard deviation in Energy industry before and after rising VAT.

Figure 3 compares the primary financial statements for the mean and standard deviation (St.d) in the energy sector before and after the VAT increase. Case data show a slight decrease in TA and TLSE after increased VAT. A drop in these two metrics could indicate cash scarcity and potentially a short-term drop in revenue. Our results confirmed that Cash CEP fell on average from 939,277.5 in 2019 to 528,862.25 in 2020, Total income fell on average from 1,000,090.75 in 2019 to 643,458 in 2020, and Total revenue also decreased from 1,082,161 in 2019 to 661,921.25 in 2020. It is consistent with Alhussain’s (2020) findings, which studied the influence of 5% VAT enforcement on Saudi banks.

Regarding shareholders’ equity, results also indicate a slight reduction between the two periods. That means the COVID-19 crisis affected companies in this sector and caused a decrease in equity due to this unusual event and the circumstances that necessitated an increase in VAT. Consequently, our results have arbitrated increases in total expenditure, which was normal due to the crisis and the increase in VAT in 2020. In addition, changes in the operational activity of enterprises increased in 2020. Still, alternatively, with Changes in Investing Activity (CIA) and Changes in Financing Act (CFA), enterprise financing decreased more in 2020 compared to 2019, which is normal due to the unusual VAT increase and COVID-19 crisis. That means there is a decrease in accounts receivable with a corresponding increase in oil inventories, a situation that must be fully assessed to improve sales. The decline in accounts receivable and a rise in oil inventory do not indicate possible concern. The rise in oil price inventories was caused by increasing production, which was foreseen and planned. Lower oil prices, rising inflation, and weaker domestic demand have all hampered GDP growth due to low-income levels in some countries. They suffered comparable negative effects on GDP growth in 2016, 2021, and 2022. Russia 1%, 0.7%, −1%, Turkey 2.9%, 3.2%, −0.6%. China 68%, 9%, Brazil 7%, India 9%, South Africa 11%, Indonesia 12%, Mexico 13%, Argentina 14%, Venezuela 15%, KSA 16-%, Thailand 17%, Philippines 18%, etc.

**Fig. 4:**
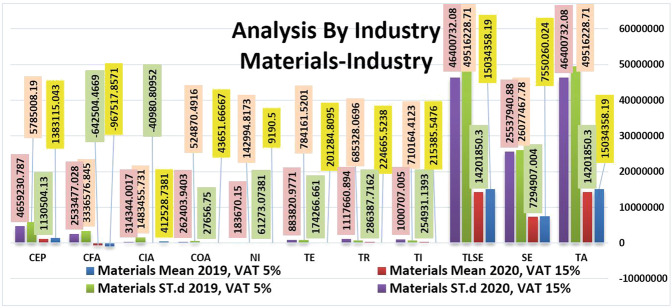
Comparison of the financial statements' key indicators in term of the average and standard deviation in Materials industry before and after rising VAT.

Figure 4 shows the results of evaluating key financial statement indicators in terms of average and St.d in the Materials Industry before and after the VAT increase. The data suggest a slight decrease in CK and TLSE after the VAT increase. This decline indicates a shortage of cash at the end of the period and a short-term decline in net income and cash. In contrast, total revenues increased slightly on average from 224,665.5238 in 2019 to 286,387.7162 in 2020, confirming the effect of the introduction of 15% VAT on net income. For clarity, we compared the average effect in the materials industry with energy in Table 2.

**Table 2:** Increase or decrease in values of financial statements’ key indicators in terms of the average between the Energy and Materials industries.

Financial statements’ key indicators	Energy (%)	Materials (%)
TA	6	6
SE	26	3
TLSE	6	6
TI	36	−18
TR	39	−27
TE	−34	13
NI	98	−567
COA	149	37
CIA	100	110
CFA	−131	34
CEP	44	18

From Table 2, the impression of the VAT and COVID-19 crisis in the materials industry in SE, TE, CFA, and CEP is less than in the energy sector. In contrast, there are significant effects on TI, TR, NI, and CIA. Net income and total sales of these companies fell by −27%. Net income in this industry fell from 9190.5 in 2019 to 6,127,307,381 in 2020 in the martial arts industry, which is more on average than in the energy industry. According to market indicators, this happened due to a decrease in demand in most industries, especially the materials industry, as most companies have closed entirely or partially since the beginning of 2020, causing demand to fall and leading to a decrease in the demand for materials.

**Fig. 5:**
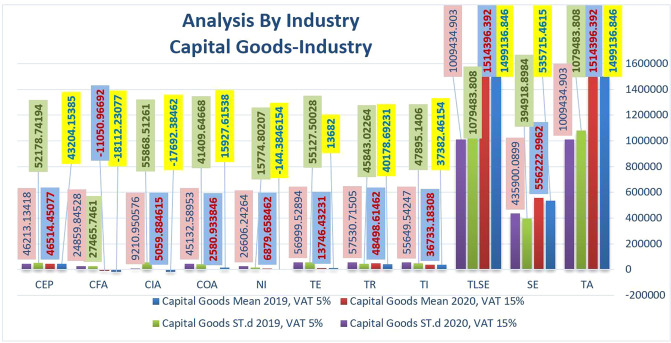
Comparison of the financial statements' key indicators in term of the average and standard deviation in Capital Goods industry before and after rising VAT.

Figure 5 highlights changes in the mean and mean St.d of critical financial indicators in the capital goods industry. On average, total assets in this industry increased twice before increasing in 2020 due to the increase in VAT due to COVID-19. The COVID-19 pandemic has been felt in worldwide commerce since three years ago, with trade shrinking and destroying the world. The international network and regional production have also taken a hit due to the disruption of global supply chains. Despite the earlier outbreak, global trade is still experiencing difficulties due to various trade issues, including the US–China trade war. There was a time of dishonesty among crucial players worldwide, which increased during and after the pandemic. As a result, some countries are moving closer to worldwide destruction or implementing a more secure state of protection against future shocks. Therefore, in the post-pandemic period, countries are expected to renew their essential support and increase their reliance on local production. For example, Japan and KSA pay cash incentives to their companies to ship their production networks home.

Reassessing the capital goods sector, the data in Table 3 show a slight decrease of 1% on average in TA and TLSE in 2020, lower than that of the energy and materials sectors. However, in this industry, the positive net income increased significantly in 2020 to 4865% compared to 2019. That means the demand for these goods will increase in 2020, as we know that material manufacturing is a tangible asset that a company wants to use to manufacture products that will be sold to customers. This rise is reflected in NI, COA, CIA, and CFA increases of 84, 129, and 39%, respectively.

**Table 3:** increase or decrease in values of financial statements’ key indicators in terms of the average between Energy, Materials, and Capital Goods industries.

Financial statements’ key indicators	Energy (%)	Materials (%)	Capital Goods (%)
TA	6	6	−1
SE	26	3	−4
TLSE	6	6	−1
TI	36	−18	2
TR	39	−27	−21
TE	−34	13	0
NI	98	−567	4865
COA	149	37	84
CIA	100	110	129
CFA	−131	34	39
CEP	44	18	−8

**Fig. 6:**
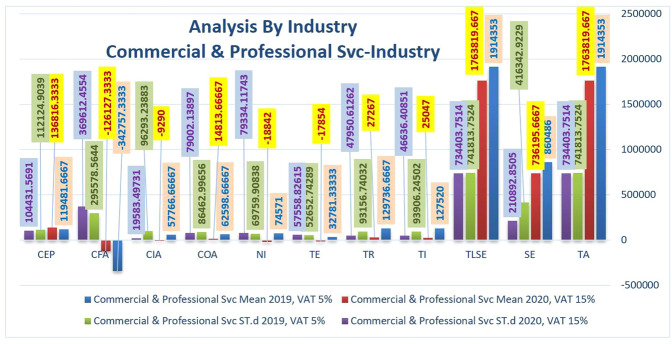
Comparison of the financial statements' key indicators in term of the average and standard deviation in Commercial & Professional Svc industry before and after rising VAT.

Figure 6 reflects the variance in mean and ST.d between key financial indicators in the business and professional services industries over the two years under study. Almost all the indicators were down slightly in 2020 when we compared them to 2019. That means this industry was not one of those industries containing high-demand materials during the COVID-19 lockdown, and the rise of VAT has negatively affected this sector. As staffing shortages continue, business and professional services firms are still battling for a return to work. Several intend to open their offices, hoping production can reproduce at pre-epidemic levels. As shifts are at the forefront of strategic planning, many companies focus on how spending more on technology can reduce inefficiency, increase operating revenue, and defend their businesses against the growing threat of bankruptcy.

In Table 4, we discovered that when we compared the averages of commercial and professional industries Svc with energy, materials, and capital goods, this industry was less affected on average in all indicators except CEP, which was severely affected. The epidemic monitoring model has accelerated the chances of further downsizing as many Commercial & Professional Svc companies have laid off employees and created vacancies over the last year. According to the “*Aderant Business of Law and Legal Technology Survey*,” conducted at the end of 2020, the ten-year decline will persist, with many companies constantly implementing remote or flexible operating measures.

**Table 4:** increase or decrease in values of financial statements’ key indicators in terms of the average between Energy, Materials, Capital Goods, and Commercial & Professional Svc industries.

Financial statements’ key indicators	Energy (%)	Materials (%)	Capital Goods (%)	Commercial & Professional Svc (%)
TA	6	6	−1	8
SE	26	3	−4	14
TLSE	6	6	−1	8
TI	36	−18	2	80
TR	39	−27	−21	79
TE	−34	13	0	154
NI	98	−567	4865	125
COA	149	37	84	76
CIA	100	110	129	116
CFA	−131	34	39	63
CEP	44	18	−8	−15

**Fig. 7:**
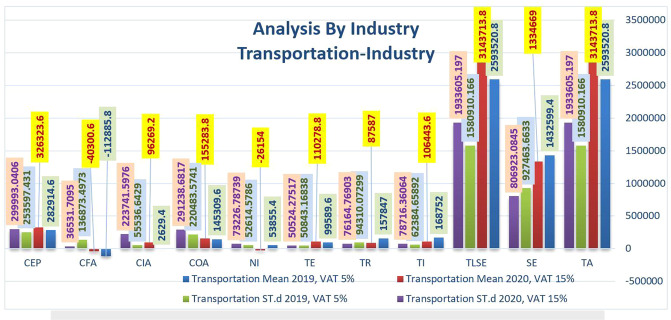
Comparison of the financial statements' key indicators in term of the average and standard deviation in Transportation industry before and after rising VAT.

Figure 7 shows an increase in total assets, TLSE, and total revenue in 2020. Conversely, a decrease in netــincome’s cash-end CEP in 2020 compared to 2019 in the transportation sector confirmed the negative impact of VAT’s increase in 2020. In addition to these pressing issues, the COVID-19 pandemic tarnishes the image of uncertainty in some parts of the global transport industry, particularly in the aviation industry. In contrast, KSA’s transport network is poised to play a vital role in the economy in the coming years. Refining Logistics and Transport infrastructure is a key objective of the “National Industrial Development Program and Logistics Program*”*, with the first victory accelerating the flow of goods into ports. A joint, multi-stakeholder effort is needed to make meaningful progress. With new bridges and roads planned for “Bahrain and Egypt, Saudi Arabia*”* also demonstrates its intention to use its territory as a transport hub connecting nations and continents in the region. Therefore, Fig. 7 shows the increases in total assets in 2020 as the Kingdom needs to realize its strategic plan to invest in this sector.

On average, Table 5 highlights that the transportation sector was heavily impacted in TA, TLSE, TI, and CIA compared to the abovementioned sectors. For example, the passenger load factor in air transport dropped to 65.1% system-wide in 2020 from 82.5% in the prior year. The region with the highest loss in passenger traffic, with a 71.55% decrease in passenger revenue per kilometer compared to 2019, is the *Middle East*, followed by the *EU* (−69.7%) and *Africa* (−68.5%).

**Table 5:** increase or decrease in values of financial statements’ key indicators in terms of the average between Commercial Materials, Energy, Capital-Goods, & Professional Svc and Transportation industries.

Financial statements’ key indicators	Energy (%)	Materials (%)	Capital Goods (%)	Commercial & Professional Svc (%)	Transportation (%)
TA	6	6	−1	8	−21
SE	26	3	−4	14	7
TLSE	6	6	−1	8	−21
TI	36	−18	2	80	37
TR	39	−27	−21	79	45
TE	−34	13	0	154	−11
NI	98	−567	4865	125	149
COA	149	37	84	76	−7
CIA	100	110	129	116	−3561
CFA	−131	34	39	63	64
CEP	44	18	−8	−15	−15

In contrast, the transport system has had many successes during 2020, the most significant of which has been increased road safety and the Kingdom’s global leadership in interconnecting the road network and the opening of King Abdulaziz International Airport in Jeddah. The Kingdom’s ports have also seen leaps and boundaries in the past year, including signing allocation and operation contracts and developing sectoral legislation. In addition to significant achievements of the Public Transport Authority, including regulating the sector, it also launched a new public fare identity.

The General Authority for Statistics (2021) said that the GDP in KSA noted a negative natural growth rate of 4.1% in 2020 compared to 2019. This negative growth is primarily due to the shrinkage of the oil industry by 6.7%. Also, a negative growth percentage of 2.3% was noted in non-oil industries. The privateـــsector fell by 3.1%, and the publicـــsector also recorded a negative percentage growth of 0.5%. In our results, we support the conclusions of the General Authority for Statistics (2021) using different data and analysis, as we find that almost all key financial statement indicators declined in 2020, except for some of them showing positive growth in 2020, as shown in Table 6 and figures in “Supplementary Information, Appendix1”. On December 31, 2020, the ten sectors declined in contrast to the growth of the rest, and the decline was controlled by “long-term goods” at 0.77%, tracked by “the luxury goods fragmentation” at 0.38%, and investment and finance up at 0.33%. In comparison, the increase was controlled by “applications and technical services” at 3.2%, followed by “insurance” at 1.4%, and “commercial and professional services” at 0.6%. The highest turnover was “basic materials” at about 17%, with a value of 1.3 billion riyals, followed by “insurance” at 13%, with a value of 1 billion riyals, and capital of production at about 12%, with a value of 919 million riyals.

**Table 6:** increase or decrease in values of financial statements’ key indicators in terms of the average between all industries.

Financial statements’ key indicators	Energy (%)	Materials (%)	Capital goods (%)	Commercial & professional Svc (%)	Transportation (%)	Consumer durables & apparel (%)	Consumer services (%)	Media and Entertainment (%)	Retailing (%)	Food & staples retailing (%)	Food & beverages (%)	Health care equipment & Svc (%)	Pharma, Biotech & Life Science (%)	Telecommunication services (%)	Utilities (%)	Real Estate Mgmt & Dev’t (%)
TA	6	6	−1	8	−21	10	3	8	4	0	−2	−9	7	0	−5	−5
SE	26	3	−4	14	7	9	−7	−31	18	7	−8	−8	16	−3	5	3
TLSE	6	6	−1	8	−21	10	3	8	4	0	−2	−9	7	0	−5	−5
TI	36	−18	2	80	37	31	52	−6	64	17	−9	−38	−15	−4	17	58
TR	39	−27	−21	79	45	5	52	1	59	17	−11	−37	−26	−3	13	56
TE	−34	13	0	154	−11	−13	26	11	11	6	2	−23	−6	1	11	−6
NI	98	−567	4865	125	149	247	152	13	131	55	−110	−54	−108	−23	19	281
COA	149	37	84	76	−7	−100	932	−190	−29	−14	93	−121	126	183	35	98
CIA	100	110	129	116	−3561	−665	8594	63	−168	−1149	1160	−81	66	73	198	213
CFA	−131	34	39	63	64	175%	−496	6564	9	−3	45	−29	59	45	27	−624
CEP	44	18	−8	−15	−15	−55	−7	36	−27	−60	3	−14	9	34	−57	−20

**Fig. 8:**
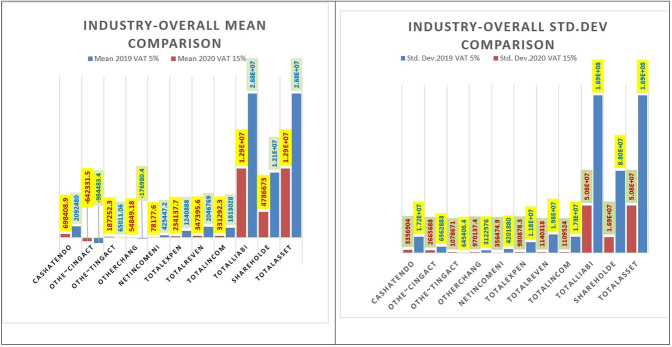
Increase or decrease in values of financial statements' key indicators in term of the average between all industries.

Figure 8 explains the implications of all comparisons of key financial statement indicators in terms of average and St.d across the sample examined before and after the VAT increase. Results generally suggest a significant decline in TA and TLSE after the VAT increase and during the COVID-19 crisis. Suggests a severe cash crunch problem and a short-term sales decline at Saudi companies. Our results confirm that the overall decrease in CEP averaged from 2,092,480 in 2019 to 698,408.9 in 2020. Further, the total TI decreased on average from 1,813,028 in 2019 to 331,292.3 in 2020, and the total TR also decreased from 204,508.9 to 2046 in 2020, supporting the results of a previous study by Alhussain (2020). However, SE’s overall results represent a similarly significant decline between the two periods. It is suffering from a decline in equity due to the unusual COVID-19 crisis and an increase in VAT. Results display an average overall increase in TE, typically due to the crisis and the increase in VAT in 2020. In addition, there was an overall increase in the authenticity of companies in 2020, but conversely, this is due to an unusual increase in VAT and the COVID-19 crisis. That signals a decline in receivables with a corresponding increase in inventories, a situation that needs to be fully assessed to increase sales.

### DiD approach analysis

Previous studies have used various valuation methods to assess VAT, including DiD based on constant cross-sections, proportional analysis, and panel-based DiD (Saez et al., 2012). This article discusses DiD based on time series. Recognition usually results from adjustments to VAT derogations made by tax restructuring. Concerns about the endogeneity of expected tools for the studied IVs and log variation in VAT have raised suggestions for modifying the standard DiD estimation model to address this endogeneity. In this regard, the average model is used to denote weakly stationary-stochastic time series in terms of multinomials. This model is regularly discussed as the ARMA-(*p*,*q*)-model. ARIMA is suitable for one-dimensional models with time-dependent instabilities. ARIMA conforms to the model of a dependent variable (DV) on IVs, where turbulence can follow a linear-autoregressive-moving-average description of ARMA.

**Fig. 9:**
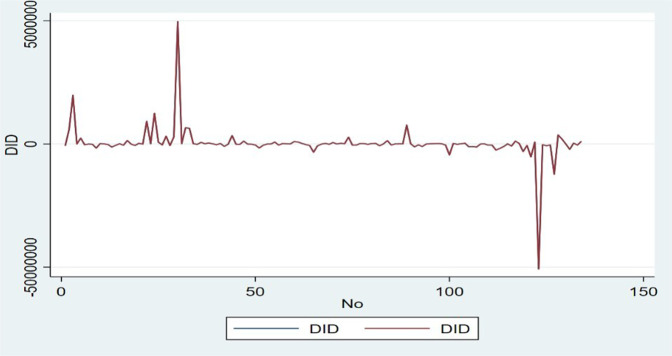
The data and logarithms first-difference.

DV and IVs may differ or be seasonally disparate to a slight degree. Once IVs are involved in the description, these models are habitually named ARMAX models; and once IVs are not recognized, they are reduced to “Box’s Autoregressive Integrated Moving-Average—Jenkins” models in DV. Likewise, most periodic ARMAX & ARIMA models may also be appropriate. Non-existent data were allowed and identified through the recommended Kalman methods (Harvey, 1990, 1993), which we used in this paper. Next, test the first difference of DiD, choosing a variance model in natural logarithm to smooth the change in different series. The first difference in data and logarithms is shown in Fig. 9.

**Fig. 10:**
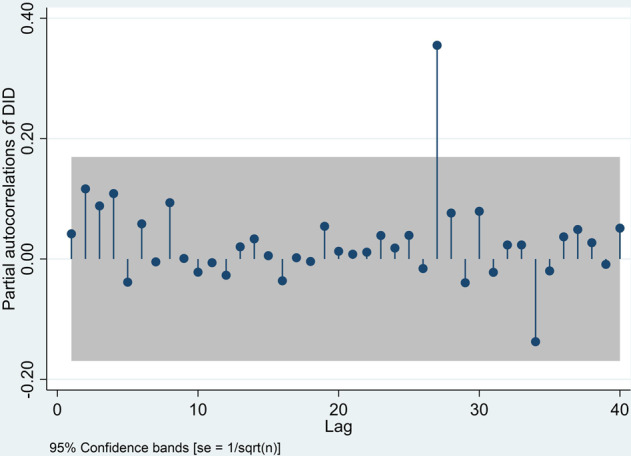
First order differential autocorrelation.

**Fig. 11:**
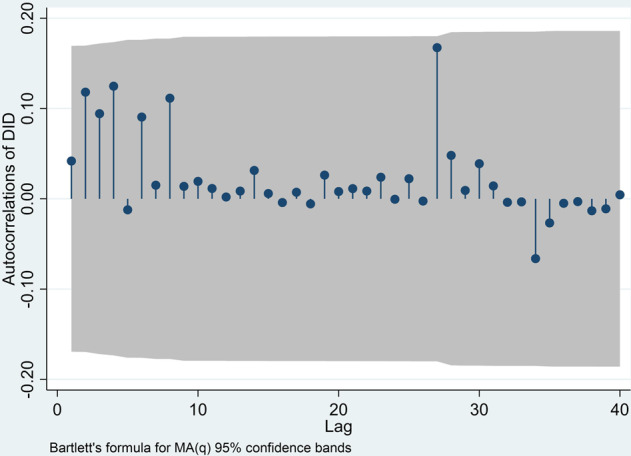
First order differential partial autocorrelation.

Based on autocorrelation, incomplete autocorrelation, and the outcomes of the initial assessments, ARMA patterns were recognized in a log discriminant series. Augmented-Dickey–Fuller (ADF) test results are shown in Table 7: Dickey–Fuller = −7003, Lag Order = 1, *p*-value = 0.000. Thus, H0 was rejected. Therefore, the self-selection hypothesis is valid, and it tends not to be stable enough that time series *d* = 1 is stationary in terms of mean and difference. In this manner, there is no need to differentiate time series further, and then the accepted discriminant requirement is *d* = 1 for ARIMA model *p*, *d*, *q*. This test allows for more means to improve ARIMA model, namely, discovering suitable values of *p* in autoregression and *q* in Moving Average (MA) in the research model. Note: In Fig. 10, the gray zone signifies 95% assurance interval for MA(*q*) model-Bartlett’s formula. Note: In Fig. 11, the gray zone signifies 95% assuredness interval for ARMA (*p, q*) model-Bartlett’s formula (SE = 1/*n*).

**Table 7:** ADF test.

Test statistic	1% Critical value	5% Critical value	10% Critical value
*Z*(*t*) −7.003	−2.356	−1.657	−1.288
*P*-value for *Z*(*t*) = 0.0000			
**D.DID Coef.**	**Std. Err.** ***t***	***P*** **>** ***t***	**[95% Conf. Interval]**
DID			
L1. −0.8455129	0.1207304 −7.00	0.000	−1.084381 −0.6066449
LD. −0.1165448	0.0872158 −1.34	0.184	−0.2891032 0.0560137
_cons 218,596.2	596,184.2 0.37	0.714	−960,968.9 1,398,161

Figure 9 shows that the autocorrelation study remains within stretch at all factor lag levels with 95% accuracy. Figure 11 shows that the preliminary autocorrelation study likewise fell outside 95% precision range for β28CFAb delay. Figures 9 and 10 outwardly show that this sequence’s autocorrelation capacity and fractional autocorrelation capacity are sought within the scope of least delay demand, and *p* and *q* are close to 0. Accordingly, we developed an ARIMA *p*, *d*, *q* model to estimate VAT. As a result, we developed “ARIMA *p, d, q* model”, which demands a differential ARIMA model according to AIC & BIC lowest data standards to control the precise values of *p* & *q*. Box et al. (2015), the creators of this time series research, argued that *p* ≤ 2 and *q* ≤ 2 would suffice as a rule.

Larger *p*-max & *q*-max values, on the other hand, can be chosen to advance the precision of the model indicator, which is predictable with the smallest order-picking standard. Mentioning ARIMA models developed by a large number of past scientists, we found that under normal conditions, *p*-max and *q*-max would not exceed the 1st order. *p* and *q* mixtures shown in Table 8, where the two parameters have a place in set D1, show that *p* = 1, *d* = 1, and *q* = 0 gave the lowest values of AIC and BIC; therefore, ARIMA 1,1,0 is the ideal model. ARIMA 1,1,0 model in time series investigation works well with time delay forecasting. We used Stata programming to determine the parameters of ARIMA 1,1,0 model and employed the maximum-likelihood evaluation method.

**Table 8:** ARIMA (1,1,0) regression.

(2019) Before Imposing VAT and COVID-19				(2020) After Imposing VAT and COVID-19			
	D.DID	Coef.	Semi-robust Std. Err.	*z*	*P* > | *z*|	[95% Conf. Interval]	
	DID						
2019	β1TAb						
	D1.	2.003555	0.0053412	375.11	0.000	1.993086	2.014024
2020	β2TAa						
	D1.	−2.003894	0.00527	−380.24	0.000	−2.014223	−1.993565
2019	β4SEb						
	D1.	1.005083	0.0065585	153.25	0.000	0.9922285	1.017937
2020	β5SEa						
	D1.	−1.005635	0.0068167	−147.53	0.000	−1.018995	−0.9922743
2019	β13TRb						
	D1.	1.608245	0.1463879	10.99	0.000	1.32133	1.89516
2020	β14TRa						
	D1.	−2.20014	0.066123	−33.27	0.000	−2.329739	−2.070541
2019	β16TEb						
	D1.	0.889937	0.0441238	20.17	0.000	0.8034558	0.9764181
2020	β17TEa						
	D1.	−0.822157	0.0543897	−15.12	0.000	−0.9287588	−0.7155552
2019	β19NIb						
	D1.	0.8353486	0.0554294	15.07	0.000	0.726709	0.9439881
2020	β20NIa						
	D1.	−0.7932042	0.0664407	−11.94	0.000	−0.9234255	−0.6629829
2019	β22COAb						
	D1.	1.062811	0.0087632	121.28	0.000	1.045635	1.079987
2020	β23COAa						
	D1.	−1.03255	0.0148645	−69.46	0.000	−1.061683	−1.003416
2019	β25CIAb						
	D1.	1.00211	0.0066044	151.73	0.000	0.9891656	1.015054
2020	β26CIAa						
	D1.	−1.002831	0.0123837	−80.98	0.000	−1.027103	−0.9785595
2019	β28CFAb						
	D1.	0.9762722	0.0063361	154.08	0.000	0.9638536	0.9886908
2020	β29CFAa						
	D1.	−0.9783687	0.006986	−140.05	0.000	−0.992061	−0.9646765
2019	β31CEPb						
	D1.	1.026975	0.0151023	68.00	0.000	0.9973752	1.056575
2020	β32CEPa						
	D1._cons	−1.011331	0.0149383	−67.70	0.000	−1.04061	−0.9820525
		108.9319	1250.183	0.09	0.931	−2341.381	2559.245
	ARMA						
	ar						
	L1.	−0.558811	0.1393543	−4.01	0.000	−0.8319404	−0.2856816
	/sigma	22,514.27	2571.836	8.75	0.000	174,73.57	27,554.98

*Notes*: ARMA, auto-regressive moving-average-model; D1 to L1, the volume of lag (1–1-0). The examination of the difference in contradiction of 0 is 1-sided, and 2-sided certainty interval is shortened at 0.

ARIMA = DID β1Tab β2TAa β4SEb β5SEa β13TRb β14TRa β16TEb β17TEa β19NIb β20NIa β22COAb β23COAa β25CIAb β26CIAa β28CFAb β29CFAa β31CEPb β32CEPa, ARIMA(1,1,0) vce (robust).

Note: 2020-D.β8TLSEa&2019-D.β7TLSEb dropped because of collinearity. 2020-D.β11TIa &2019-D.β10TIb dropped because of collinearity.

Table 8 shows the estimation of ARMA model with first-order (AR1) autoregressive terms with the initial log differential DV of DiD and exogenous initial log differential IVs of VAT (D1. from B1 to B32, 2019–2020) and the initial differential logarithm of the unemployment rate (D1. B1 to B32). The “vce*”* choice is involved, which produces semi-robust standard errors. We fitted a model with 2 lags for the AR portion, ar (1), and 1 lag for the MA part, ma (1). Otherwise, we can have captured the returns-ARIMA (1,1,0); here, the initial digit designates that we need to insert 2 lags for the AR part, and the subsequent digit indicates that we need to add the order of integration here equal to 0. The third digit indicates that we want to insert a lag for the MA part. In the primary output part, we discover info concerning the optimization process, with the repetitions of the algorithm intended to exploit the logـــlikelihood role. The intersection is performed in 19 stages and stops at log-likelihood value of −1521.819.

We are further notified that the estimated sample contains 133 annotations and that the model is generally statistically expressive, as indicated by Wald test. Table 8 delivers strictures, standard errors, and a *t*-test for the statistical substance of parameters for *P* > | *z* | and a 95% confidence interval. From the outcomes of ARMA model mentioned above, we realize that the expression of AR1 disruption is statistically meaningful (beta = 0.455, *p* < 0.01). L1 is negative, and a statistically important approximation of −0.558 for the autoregressive constant designates that there is a significant amount of optimistic autocorrelation in this sequence. Both Figs. 8 and 9 show no autocorrelation of ARMA residues with AR1 deterioration conditions. We used a general C–H Test in Table 9 to distinguish autocorrelation (Titus, 2021).

**Table 9:** C–H test.

HQ: *q* = *Q* (seriallyــuncorrelated)				H0:	*q* = *Q* (seriallyــuncorrelated)		
HA: S.C. present at range specified				HA:	S.C. present at	Lag-specified	
lags	chi2	df	*p*-val	lag	chi2	df	*p*-val
1- 1	2.439	1	0.1183	1	2.439	1	0.1183
1- 2	13.900	2	0.0010	2	5.455	1	0.0195
1- 3	13.925	3	0.0030	3	0.093	1	0.7599
1–4	14.065	4	0.0071	4	0.511	1	0.4746

Test robust to heteroskedasticity.

### Critical thoughts discussions

In this section, we interpret statistical results through an in-depth analytical discussion that enables us to answer, “So what?” with further improvements to make it easier for readers to understand. Furthermore, empirical findings have been fully demonstrated, analyzed, compared with previous studies, and critically evaluated using a comprehensive extended analysis.

Outcomes show that a severe VAT increase has a considerable positive and negative influence on inter-industry volatility in corporate financial reporting metrics. We address the following hypotheses about the causes of the opposite effect of a VAT decrease/increase in different sectors: Initially, regarding the opposite effect of a VAT decrease in other sectors, the price of customer goods & services may be depressed when government accumulates a fresh source of income. Depressing the price of customer goods & services with VAT as an option to income-tax-governments can advance residents’ living standards. An enhanced standard of living might run to augment tax incomes from fostered taxable revenue. Tax cuts for firms might inspire economic progress by permitting organizations to invest further in their functions. For example, several countries decreased corporate taxes throughout the 2008–2009 collapse to encourage economic development. In this regard, Greece in 2013 cut company taxes by 25% under its bailout program with the EU and IMF. The following year, Greece had its best economic accomplishment in years, with corporate exports and investments growing because of a cut in corporate taxes.

Reducing the VAT rate could affect numerous positive modifications in the economy. Nations with lower customer prices are wealthier than those without, owing to increased tax revenues for government activities supported by lower tax revenues. Customers waste more when prices are low, so a drop in customer prices leads to increased company sales. Discounted prices similarly enable residents to buy additional goods & services. Therefore, increased domestic demand drives economic growth by increasing residents’ spending on goods & services and generating income tax revenues. Implementing VAT preserve expands citizens’ quality of life by increasing government agencies’ revenues functioning at lesser prices and inferior tax rates at trade outlets. It is therefore recommended that all nations implement such programs, as they can simultaneously advance all sectors of the community economy.

Contrariwise, the opposite impression of a VAT increase can be observed in different sectors. When tax load cannot be fully relocated, the capital cost of advanced input VAT is taken into account, or changes in product demand are produced by the alteration in tax proportion, VAT is no longer fully neutral and is further likely to distress business performance and cost. Ormaechea and Morozumi (2019) discussed why it could matter for growth if VAT is increased. That is mainly due to specific design elements of VAT, such as exemptions and differential taxes, which can cause ineffective distribution of resources. Even though theory frequently proposes that consumption taxes in aggregate may not directly misrepresent the investment decisions of optimizing agents due to disparities in income taxes. VAT charged on inputs is not refunded or credited, but exemptions indicate that no tax is charged on sales, whereas net VAT taxes only sales and allows listed corporations to request that tax be charged on their inputs. Arguing that tax exemption distorts firms’ selection of entry and creates a component of construction tax (Crawford et al., 2010; Keen, 2013; Cnossen, 2020), they probably compromise the effective distribution of sources, potentially leading-negative growth outcome.

In addition, the application of differentiated tax rates that reduce tax rates on certain services and goods might similarly have a negative impact on growth. They also raise the administrative cost Ebrill et al. (2001) and distort customer elections by influencing comparative prices (Mirrlees, 2011). To sum up, increasing VAT income through increasing the tax base, reducing tax exemptions, or adopting a more uniform tax rate assembly, fewer tax rate reductions may be more conducive to growth than increasing revenue through tax increases. Standard rates apply to most taxed consumption, as the last increase in OECD countries may forgo the efficacy gain (Ormaechea and Morozumi, 2019). Dabla-Norris and Lima (2018) also examine macro-economic influences, separating the consequences of tax ratios and tax base changes. Their outcomes on the output influence of the VAT rate show that a rise in the VAT rate has a statistically significant negative influence on output in the short term.

However, sales in some industries may rise, whereas others may decline. For instance, some industry analysis charts show that the average growth in their financial statements’ key indicators is consistent with IMF programs related to diverse tax revenues over a 3-year time horizon. Generally, the IMF program is positively associated with a rise in goods & services taxation of 0.7% of GDP (Reinsberg et al., 2020). Given the impact of the universal financial crisis, it is frequently contended that there may have been a systemic change in growth forms in developed economies following the 2007–2009 global financial crisis (Acosta-Ormaechea and Morozumi, 2021). Consistent with this dispute, the median growth proportion of GDP per capita in OECD nations from 1993 to 2007 was 2.5%, compared with 1.51% from 2010 to 2018. The theoretical clarification for this outward alteration is the secular-stagnation assumption of Summers (2014).

Contrariwise, VAT may impact export performance, as a 1% reduction in export VAT in China results in a 7.2% increase in the value of permitted exports (Gourdon et al., 2022). In addition, the change in a country’s business tax to VAT reform has also encouraged organizations to expand innovation output and content (Cao et al., 2022). In Indonesia, both the introduction of electronic invoices and taxpayer compliance have had a significant positive impact on VAT revenue. Further improvements in its service to taxpayers will improve compliance and increase tax revenue, which could impact VAT revenue (Irawati et al., 2022). In addition, South Korea’s trust significantly affects cooperative VAT compliance when the discount amount increases (Kim et al., 2022). In Asia, implicit tariffs, services, and government efficiency have a meaningful positive impact on VAT revenue, while import volumes have an important negative impact.

In contrast, standard rates, fiscal shortages, effectiveness, control of corruption, the rule of law, and the rule of democracy have no substantial influence on VAT receipts (Permadi and Wijaya, 2022). Previous research has also shown that these VAT changes thus provide a perfect combination to assess the impact of changing sales and sales tax through VAT on business performance (Adhikari, 2020) and explain that the hypothetical benefits of VAT are not inherently successful. In fact, in Saudi Arabia, we expect the impact of VAT on economic output to be affected by the level of progress in a country. Therefore, the government should consider meaningful outcomes before implementing the following tax rate reform interventions in non-financial and other sectors. Furthermore, if GCC countries want to introduce or reform their VAT systems, they must ensure that the system is moderate and more impoverished for low-income households (Alavuotunki et al., 2019).

## Conclusion and remarks

This article scrutinizes the influence of new VAT enforcement on Saudi non-financial listed companies. To attain the research objective and policy implications, we compared the effects of the new VAT on different sectors of non-financial companies. Using appropriate charts, tables, and DiD analysis approach with the ARMA model, we examined the impacts of the new 15% VAT on companies. On the data front, we targeted 2019 before the new VAT and the discovery of COVID-19 and 2020 after the new VAT and during the COVID-19 pandemic. Results uncovered the consequences of an unexpected VAT escalation, raising whether they verified a real tax system within KSA. The outcomes show that a strong VAT increase significantly affects positive/negative cross-industry volatility in corporate financial reporting indicators, which is even more severe with the COVID-19 disaster.

Previous studies have shown that the hypothetical benefits of VAT do not explain its success. Strictly speaking, in Saudi Arabia, we would expect the effect of VAT on economic performance to be influenced by a country’s stage of development. According to global studies, progress status is strongly related to factors such as the informal economy, tax evasion, and tax capacity, which can mitigate the effect of strict VAT application. Furthermore, previous studies have cited that VAT is often remembered as a possible alternative to raising needed revenue and distorting taxes on public spending, particularly in developing countries. Based on that, we anticipate the same results as this study about KSA, one of the fastest emerging developing countries.

In KSA, the proposed 10% VAT increase caused significant volatility in the company’s indicators (e.g., on average, a −2.16% decrease in profitability, more inactive companies, and a higher probability of some companies going bankrupt). Such an outcome would inevitably affect unemployment and reduce tax revenue in the long run. Therefore, the Saudi Arabian government should consider results with practical implications before implementing the following tax rate reform interventions for non-financial sectors. We emphasize the importance of updating the tax system under the Saudi VAT implementation to utilize VAT assets effectively. These effects are consistent with previous research on development and taxation that maintains that the actual tax system is not admittedly regulated but one that is managed (Gillis et al., 1990; Bird and Gendron, 2007; Gordon and Li, 2009; Adhikari, 2020).

This study explains the significant policy impact that may be involved for KSA and other GCC countries considering significant VAT rate reform. In line with Mgammal’s (2021) study, this in-depth analysis supports the hypothesis that post-VAT rate hike firms are, on average, less profitable. However, Mgammal (2021) found that a 10% increase in applied VAT resulted in a −2.16% reduction in the average profitability of Saudi firms. Thus, this study confirms that VAT management and employment in Saudi Arabia have received more attention recently, particularly given previous results, which demonstrated significant revenue in terms of economic efficiency through implementing a well-implemented and well-adapted VAT system. Also, this article provides strong evidence of a significant negative impact of the upsurge in the VAT rate on corporate profitability (in the short term) and several other indicators. It is essential to document that the magnitude of the impact can be affected by abandoned reasons. In the long term, the rise in taxes is expected to increase the companies’ profitability because it raises government revenues. Afterward, government investments can increase businesses’ profitability or achieve social goals like diminishing economic inequalities, but taxation is expected only to improve business profitability.

Our article has several policy implications. VAT can replace distortionary taxes and generate much-needed income for public expenditures, particularly in developing countries. Although an external tax, a rate change can impact operating costs and business performance. An increase in the VAT rate does not merely affect businesses’ tax load and transaction costs. It similarly affects production costs, finance volume, distribution costs, and business management costs.

A country’s level of development will determine the impact of VAT on the economy. The level of development is closely linked to factors such as tax evasion, tax capability, and the informal economy, which can severely destabilize the efficiency of a VAT. Consequently, management should control products’ pricing and services through the industrial chain to evade unjustified loss of interest from weak originalities. Additionally, the findings of this article can serve as a reference for KSA to optimize its VAT system better and for other countries, e.g., GCC, to optimize their tax policies after the COVID-19 pandemic and worldwide recession. Furthermore, given the limitations of the article, further research is merited to determine the association between taxation and governance in developing countries; this is a huge topic in and of itself and requires more detailed analysis. This study only examines one sort of tax (VAT) with a small sample, so a more comprehensive and exhaustive examination of other types of taxes is needed to draw broader business conclusions about taxation as an engine of governance. The lack of studies about tax planning in developing nations, e.g., GCC, is another crucial issue that needs further investigation. Finally, in terms of analysis, researchers can use meta-analysis and plot probability charts, which we think would also work well with future research.

## Supplementary information

Appendix1:

## Data Availability

The data supporting this study’s findings are available on request from the corresponding author.

## Footnotes

1 “Gulf Cooperation Council”.

2 Statement of financial position.

3 Statement of comprehensive income.
