# Supplementary material for: Value-added-tax rate increases: A comparative study using difference-in-difference with an ARIMA modeling approach
Source: Humanit Soc Sci Commun. 2023 Mar 21;10(1):121. doi: 10.1057/s41599-023-01608-y (PMC10028327; doi:10.1057/s41599-023-01608-y)
Supplement: Supplementary file 1 — Appendix1: [file 41599_2023_1608_MOESM1_ESM.pdf]

Appendix1: The rest of industries, mean and ST.d analysis of each one separately

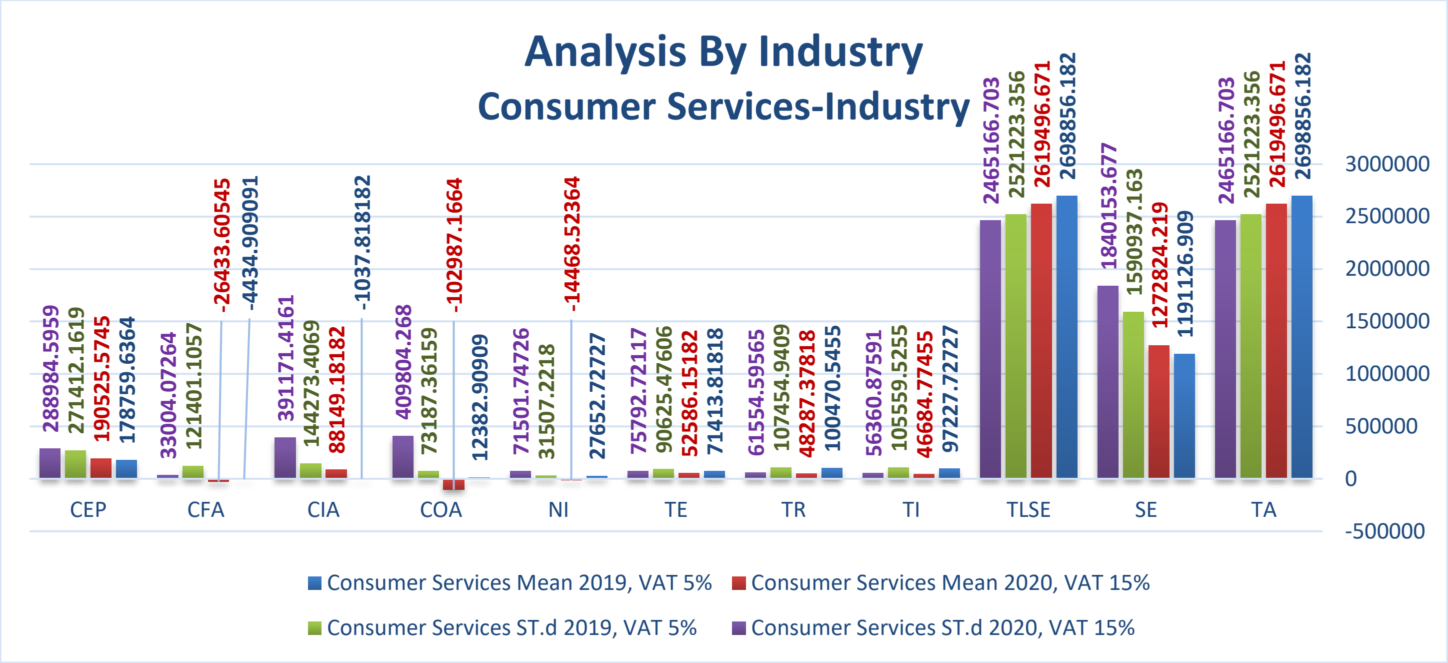

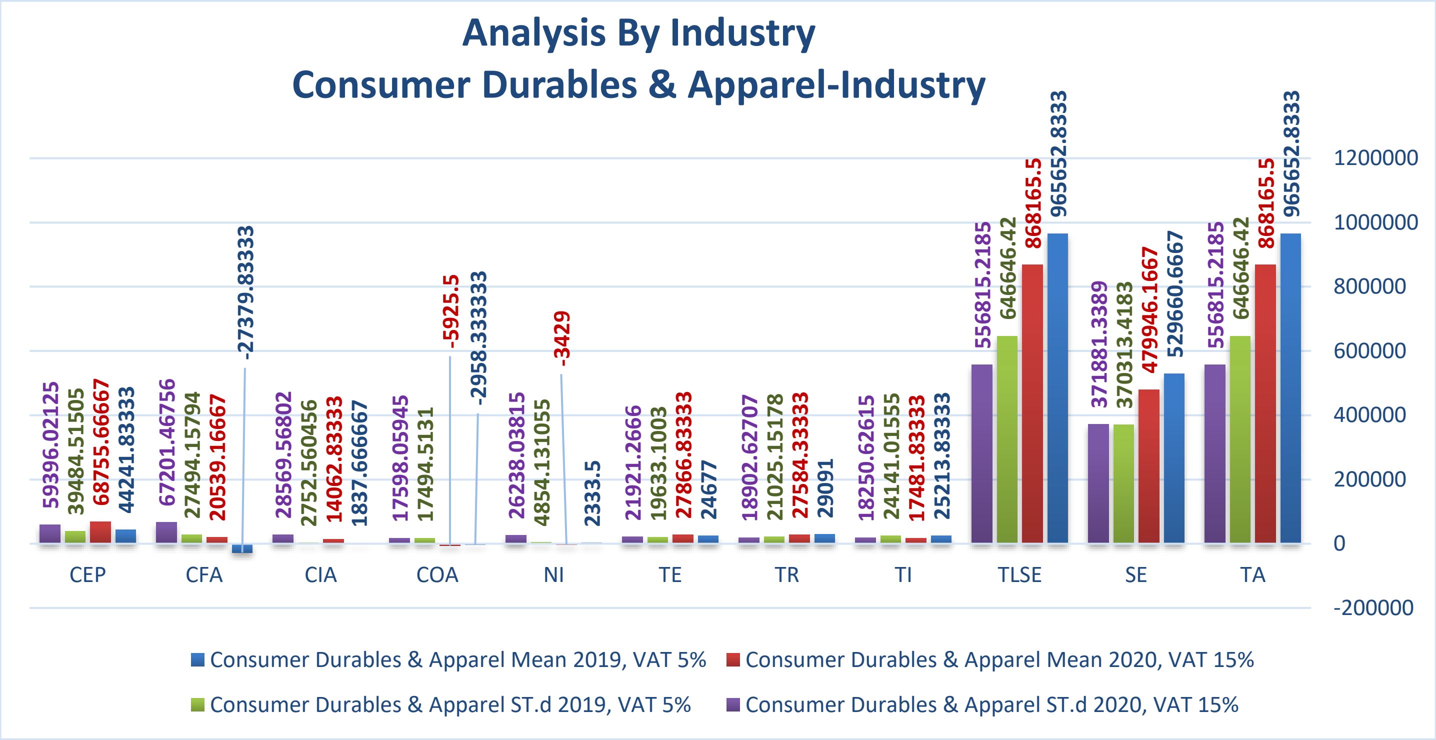

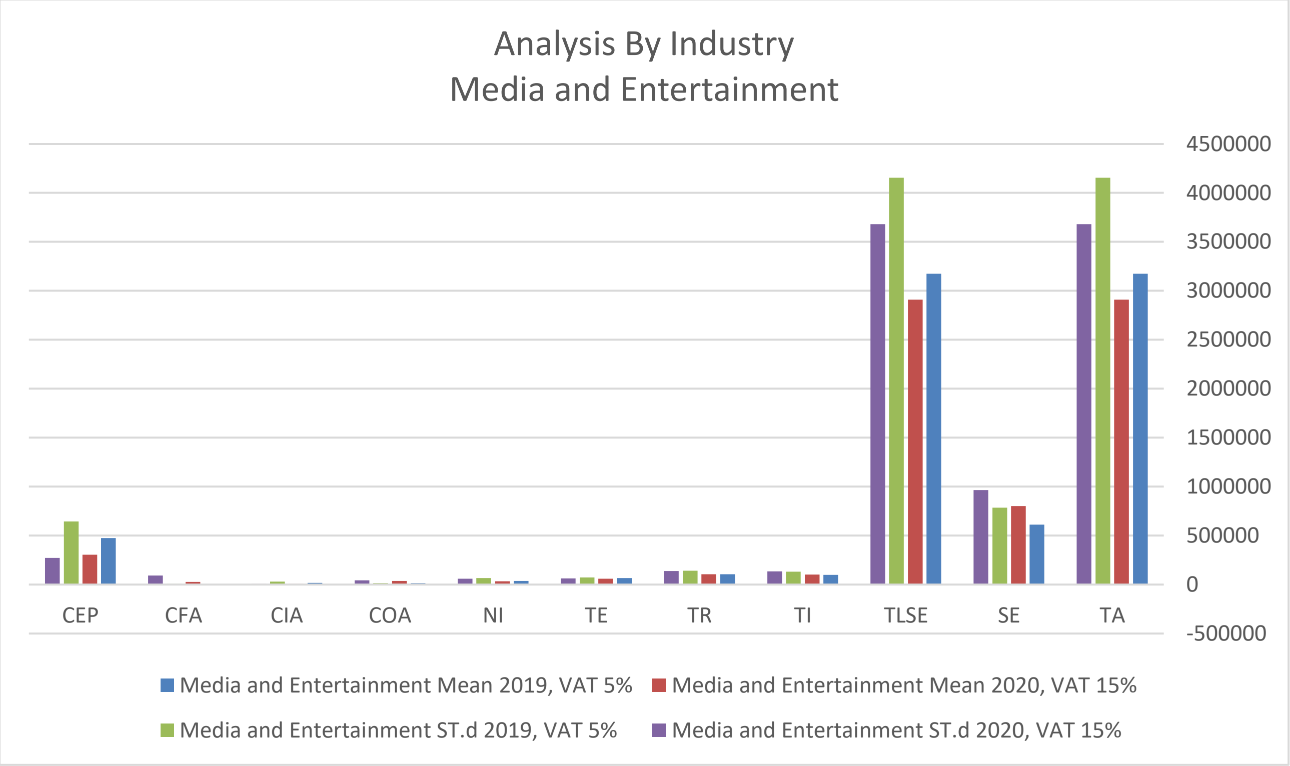

Analysis By Industry
Media and Entertainment
4500000
4000000
3500000
3000000
2500000
2000000
1500000
1000000
500000
0
-500000
CEP
CFA
CIA
COA
NI
TE
TR
TI
TLSE
SE
TA
Media and Entertainment Mean 2019, VAT 5%
Media and Entertainment Mean 2020, VAT 15%
Media and Entertainment ST.d 2019, VAT 5%
Media and Entertainment ST.d 2020, VAT 15%

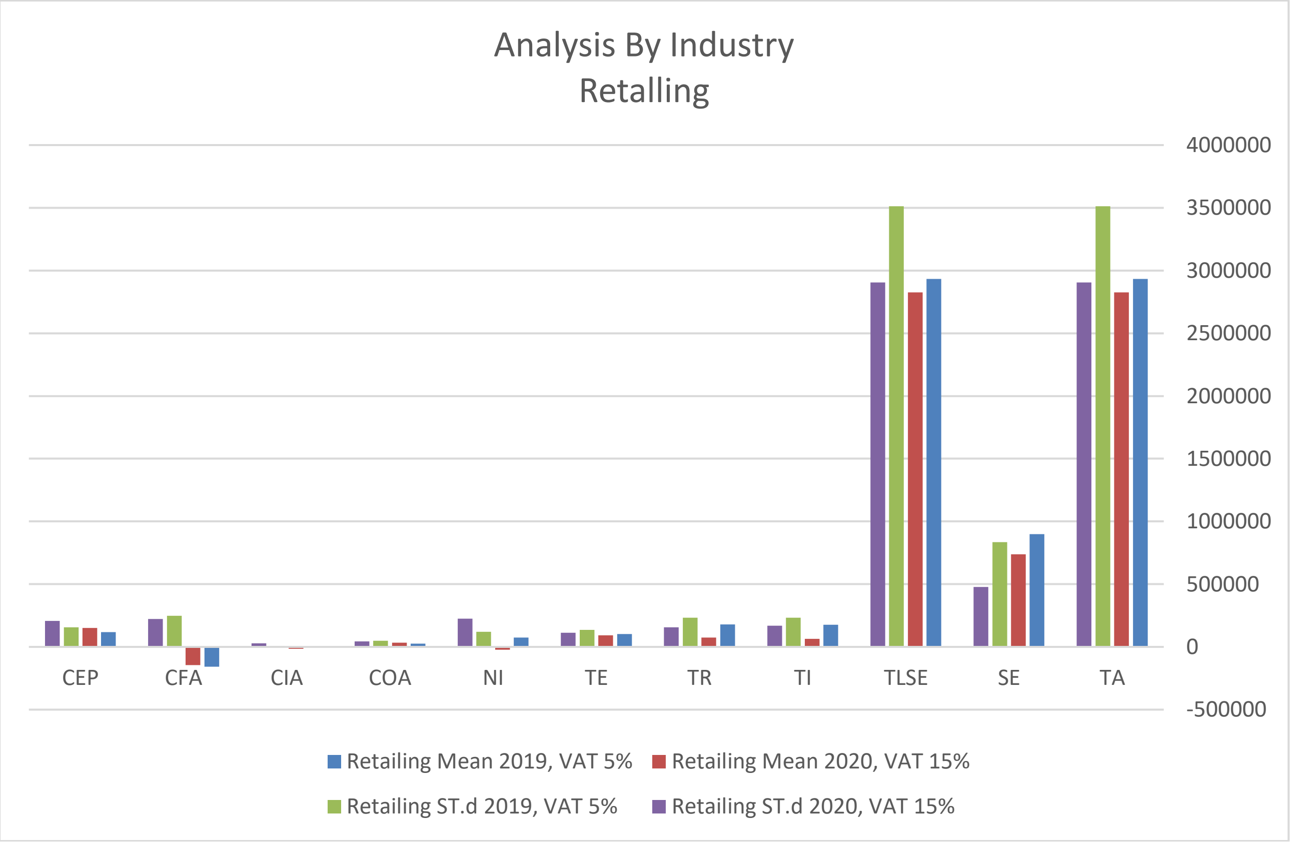

Analysis By Industry
Retalling
4000000
3500000
3000000
2500000
2000000
1500000
1000000
500000
0
-500000
CEP
CFA
CIA
COA
NI
TE
TR
TI
TLSE
SE
TA
Retailing Mean 2019, VAT 5%
Retailing Mean 2020, VAT 15%
Retailing ST.d 2019, VAT 5%
Retailing ST.d 2020, VAT 15%

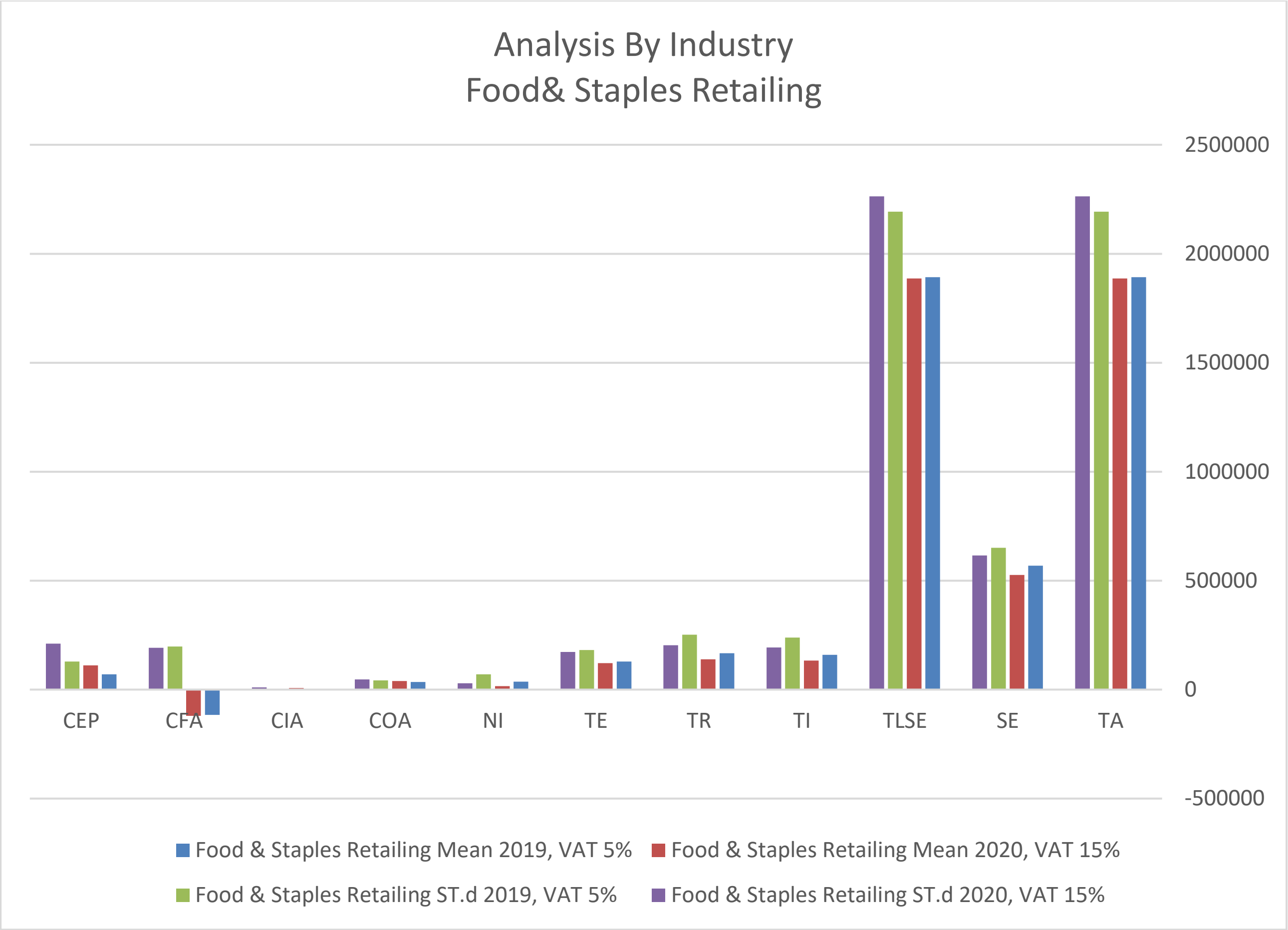
Analysis By Industry
Food& Staples Retailing
2500000
2000000
1500000
1000000
500000
0
-500000
CEP
CFA
CIA
COA
NI
TE
TR
TI
TLSE
SE
TA
Food & Staples Retailing Mean 2019, VAT 5%
Food & Staples Retailing Mean 2020, VAT 15%
Food & Staples Retailing ST.d 2019, VAT 5%
Food & Staples Retailing ST.d 2020, VAT 15%

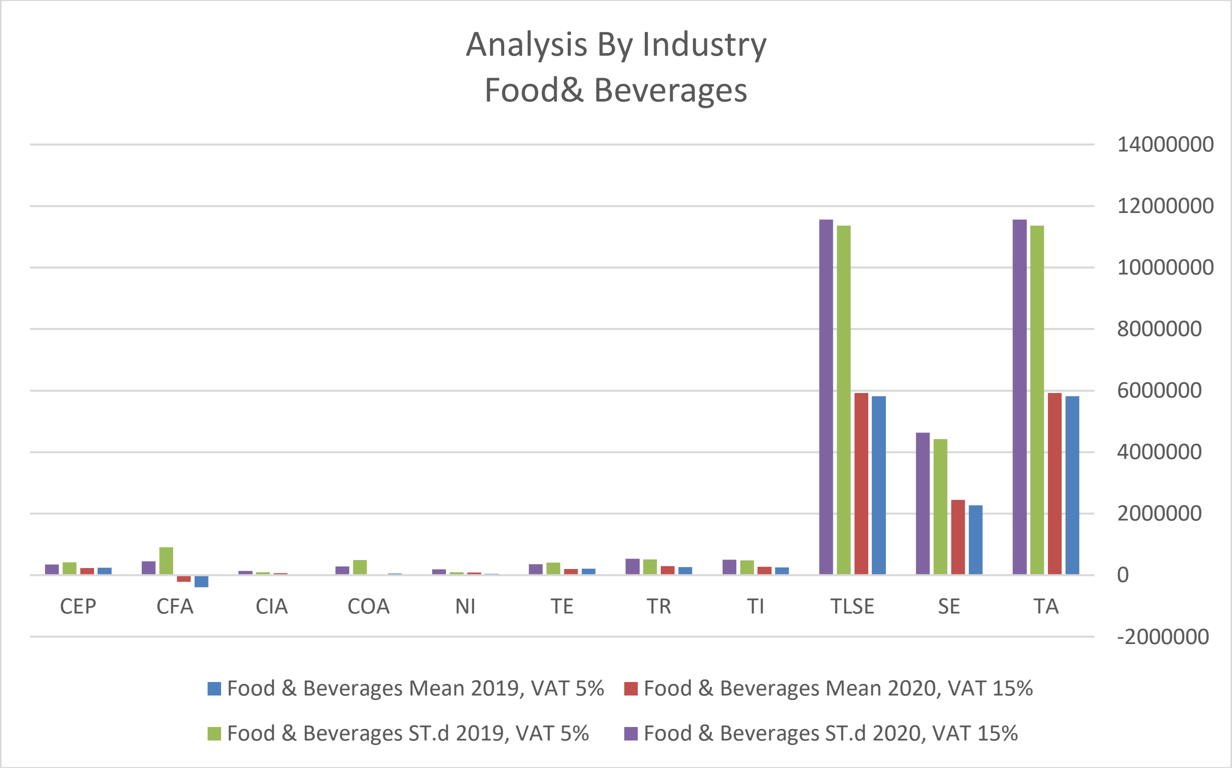
Analysis By Industry
Food& Beverages
14000000
12000000
10000000
8000000
6000000
4000000
2000000
0
-2000000
CEP
CFA
CIA
COA
NI
TE
TR
TI
TLSE
SE
TA
Food & Beverages Mean 2019, VAT 5%
Food & Beverages Mean 2020, VAT 15%
Food & Beverages ST.d 2019, VAT 5%
Food & Beverages ST.d 2020, VAT 15%

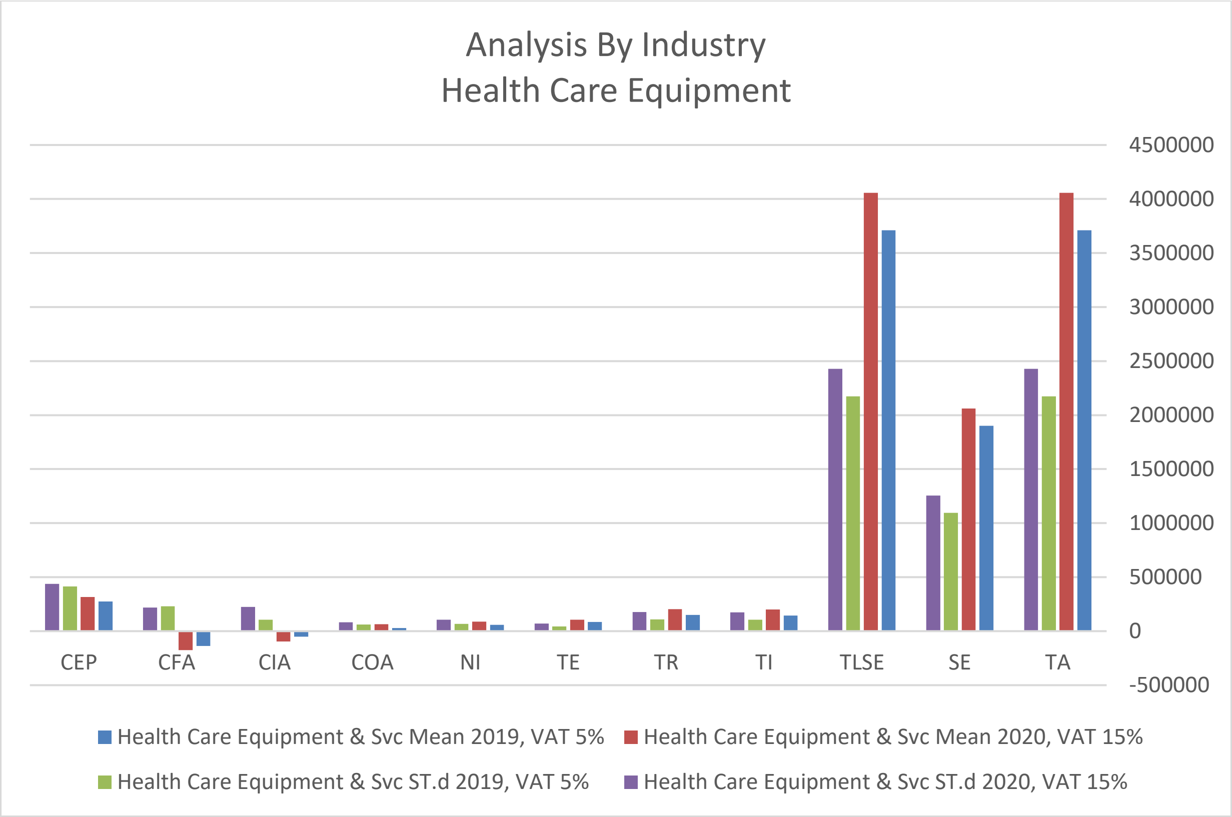
Analysis By Industry
Health Care Equipment
4500000
4000000
3500000
3000000
2500000
2000000
1500000
1000000
500000
0
-500000
CEP
CFA
CIA
COA
NI
TE
TR
TI
TLSE
SE
TA
Health Care Equipment & Svc Mean 2019, VAT 5%
Health Care Equipment & Svc Mean 2020, VAT 15%
Health Care Equipment & Svc ST.d 2019, VAT 5%
Health Care Equipment & Svc ST.d 2020, VAT 15%

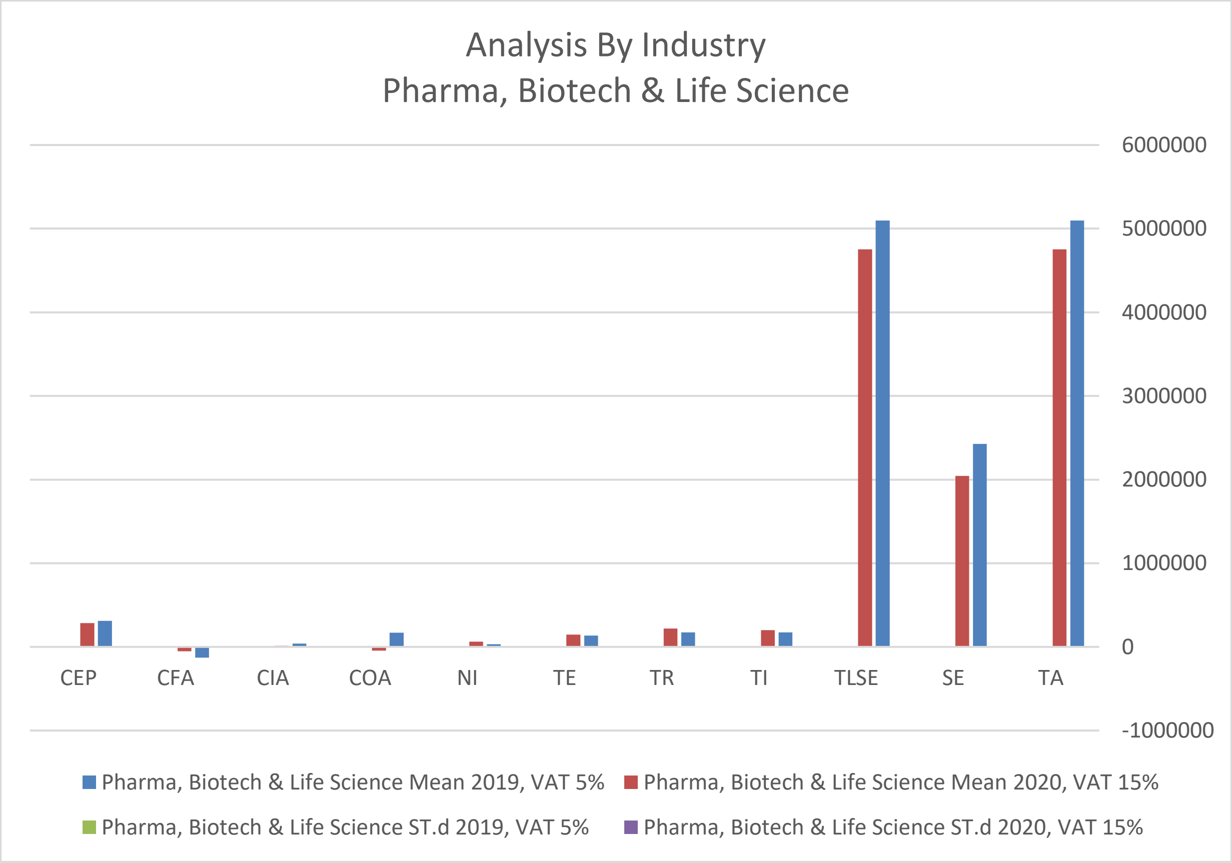
Analysis By Industry
Pharma, Biotech & Life Science
6000000
5000000
4000000
3000000
2000000
1000000
0
-1000000
CEP
CFA
CIA
COA
NI
TE
TR
TI
TLSE
SE
TA
Pharma, Biotech & Life Science Mean 2019, VAT 5%
Pharma, Biotech & Life Science Mean 2020, VAT 15%
Pharma, Biotech & Life Science ST.d 2019, VAT 5%
Pharma, Biotech & Life Science ST.d 2020, VAT 15%

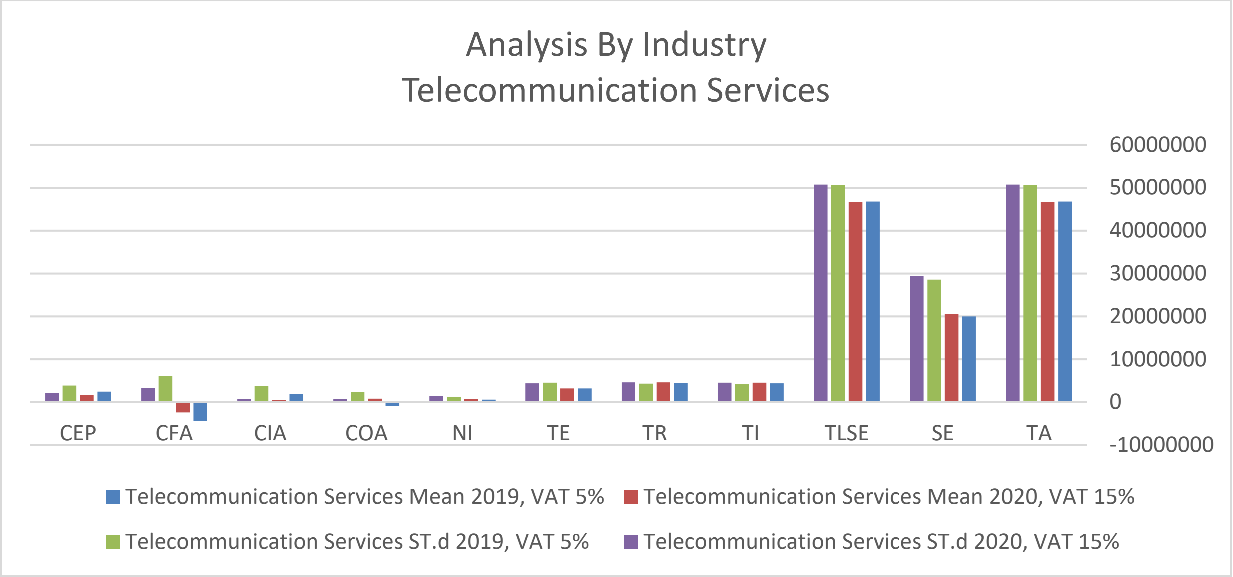
Analysis By Industry
Telecommunication Services
60000000
50000000
40000000
30000000
20000000
10000000
0
-10000000
CEP
CFA
CIA
COA
NI
TE
TR
TI
TLSE
SE
TA
Telecommunication Services Mean 2019, VAT 5%
Telecommunication Services Mean 2020, VAT 15%
Telecommunication Services ST.d 2019, VAT 5%
Telecommunication Services ST.d 2020, VAT 15%

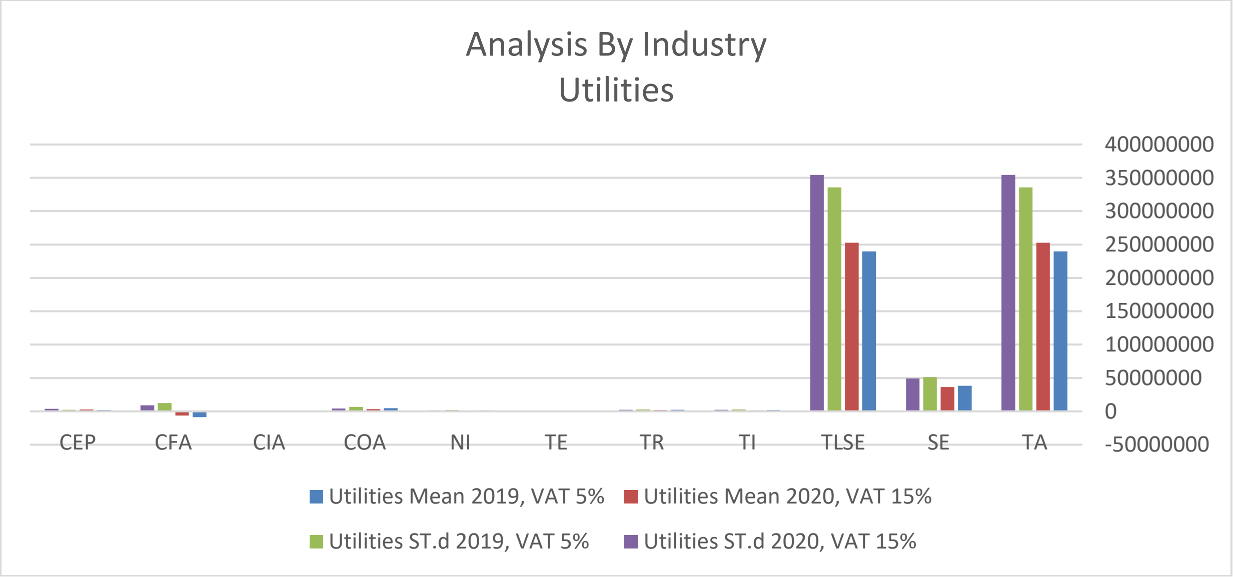
Analysis By Industry
Utilities
400000000
350000000
300000000
250000000
200000000
150000000
100000000
50000000
0
-50000000
CEP
CFA
CIA
COA
NI
TE
TR
TI
TLSE
SE
TA
Utilities Mean 2019, VAT 5%
Utilities Mean 2020, VAT 15%
Utilities ST.d 2019, VAT 5%
Utilities ST.d 2020, VAT 15%

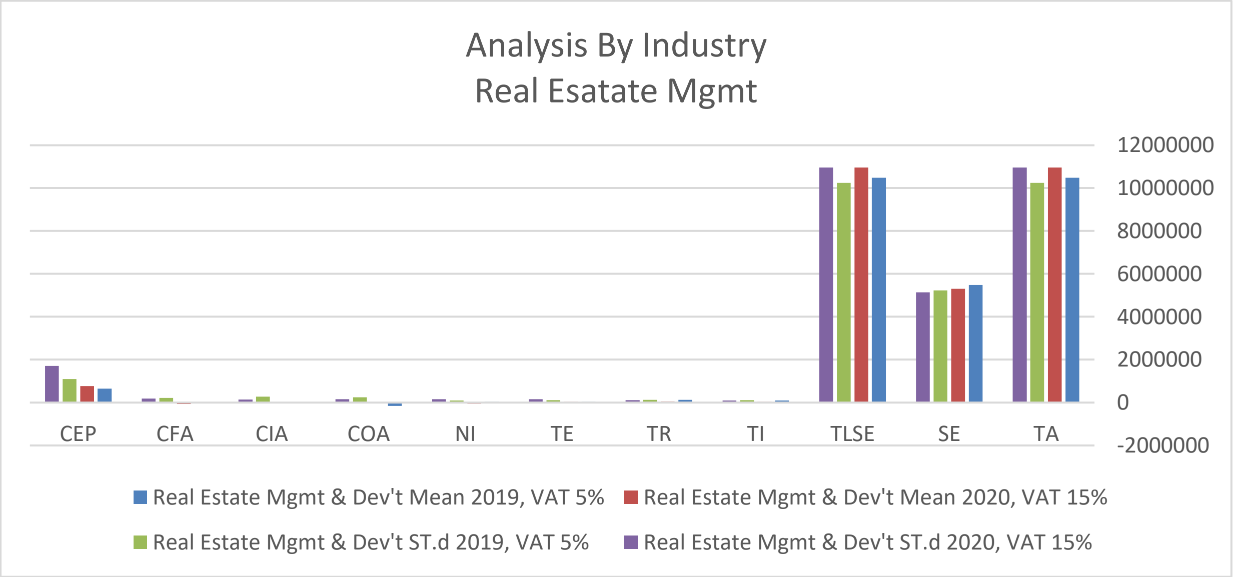
Analysis By Industry
Real Esatate Mgmt
12000000
10000000
8000000
6000000
4000000
2000000
0
-2000000
CEP
CFA
CIA
COA
NI
TE
TR
TI
TLSE
SE
TA
Real Estate Mgmt & Dev't Mean 2019, VAT 5%
Real Estate Mgmt & Dev't Mean 2020, VAT 15%
Real Estate Mgmt & Dev't ST.d 2019, VAT 5%
Real Estate Mgmt & Dev't ST.d 2020, VAT 15%
